# Prevalence and Molecular Characterization of Carbapenemase-Producing Multidrug-Resistant Bacteria in Diabetic Foot Ulcer Infections

**DOI:** 10.3390/diagnostics15020141

**Published:** 2025-01-09

**Authors:** Mohd Saleem, Soha Abdallah Moursi, Tahani Nasser Almofeed Altamimi, Mohammed Salem Alharbi, Alwaleed Mohammad Alaskar, Sahar Adly Hassan Hammam, Ehab Rakha, Ozair Ilyas Syed Muhammad, Hamoud Abdulmohsin Almalaq, Metab Nasser Alshammari, Azharuddin Sajid Syed Khaja

**Affiliations:** 1 Department of Pathology, College of Medicine, University of Hail, Hail 55476, Saudi Arabia; s.moursi@uoh.edu.sa; 2 Department of Family and Community Medicine, College of Medicine, University of Hail, Hail 55476, Saudi Arabia; ta.altamimi@uoh.edu.sa; 3 Department of Internal Medicine, College of Medicine, University of Hail, Hail 55476, Saudi Arabia; ms.alharbi@liveuohedu.onmicrosoft.com; 4 Department of Diabetes and Endocrinology, King Salman Specialist Hospital, Hail 55471, Saudi Arabia; alwaleed-alaskar@hotmail.com; 5 Department of Microbiology, Maternity and Children Hospital, Hail 55471, Saudi Arabia; sh-hammam@moh.gov.sa; 6 Laboratory Department, King Khalid Hospital, Hail 55421, Saudi Arabia; ehabrakha@yahoo.com; 7 Clinical Pathology Department, Faculty of Medicine, Mansoura University, Mansoura 7650030, Egypt; 8 Department of Surgery, King Khalid Hospital, Hail 55421, Saudi Arabia; sozairilyas@moh.gov.sa; 9 Department of Medical Supplies, Hail Health Cluster, Hail 55471, Saudi Arabia; halmalaq@moh.gov.sa; 10 Department of Infection Control, King Khalid Hospital, Hail 55421, Saudi Arabia; metab2201@gmail.com

**Keywords:** antimicrobial resistance (AMR), carbapenemase-producing organisms, diabetic foot ulcers, multidrug-resistant bacteria, *Klebsiella pneumoniae* carbapenemase

## Abstract

**Background:** Diabetic foot ulcers (DFUs) represent severe complications in diabetic patients, often leading to chronic infections and potentially resulting in nontraumatic lower-limb amputations. The increasing incidence of multidrug-resistant (MDR) bacteria in DFUs complicates treatment strategies and worsens patient prognosis. Among these pathogens, carbapenemase-producing pathogens have emerged as particularly concerning owing to their resistance to β-lactam antibiotics, including carbapenems. **Methods:** This study evaluated the prevalence of MDR bacteria, specifically carbapenemase-producing pathogens, in DFU infections. A total of 200 clinical isolates from DFU patients were analyzed via phenotypic assays, including the modified Hodge test (MHT) and the Carba NP test, alongside molecular techniques to detect carbapenemase-encoding genes (*blaKPC*, *blaNDM*, *blaVIM*, *blaIMP*, and *blaOXA-48*). **Results:** Among the isolates, 51.7% were confirmed to be carbapenemase producers. The key identified pathogens included *Klebsiella pneumoniae*, *Pseudomonas aeruginosa*, *Acinetobacter baumannii*, and *Escherichia coli*. The most commonly detected carbapenemase genes were *blaKPC* (27.6%) and *blaNDM* (24.1%). Carbapenemase-producing isolates presented high resistance to β-lactam antibiotics, whereas non-carbapenemase-producing isolates presented resistance through mechanisms such as porin loss and efflux pumps. **Conclusions:** The findings of this study highlight the significant burden of MDR infections, particularly carbapenemase-producing organisms, in DFUs. MDR infections were strongly associated with critical clinical parameters, including pyrexia (*p* = 0.017), recent antibiotic use (*p* = 0.003), and the severity of infections. Notably, the need for minor amputations was much higher in MDR cases (*p* < 0.001), as was the need for major amputations (*p* < 0.001). MDR infections were also strongly associated with polymicrobial infections (*p* < 0.001). Furthermore, Wagner ulcer grade ≥II was more common in MDR cases (*p* = 0.002). These results emphasize the urgent need for enhanced microbiological surveillance and the development of tailored antimicrobial strategies to combat MDR pathogens effectively. Given the high prevalence of carbapenem resistance, there is an immediate need to explore novel therapeutic options to improve clinical outcomes for diabetic patients with DFUs.

## 1. Introduction

Diabetic foot ulcers (DFUs) are among the most debilitating complications of diabetes mellitus, with an estimated lifetime incidence of 15–25% in diabetic individuals [1]. These chronic wounds often progress to severe infections, osteomyelitis, and lower limb amputations, significantly contributing to global morbidity, mortality, and healthcare expenditures [2]. DFUs are the leading cause of nontraumatic lower limb amputations, underscoring their clinical and socioeconomic impact. The global diabetes epidemic, affecting over 400 million individuals, is directly fueling the rising incidence of DFUs and their associated complications [3].

The pathogenesis of DFUs involves a multifactorial interplay between neuropathy, peripheral vascular disease, and impaired wound healing, all exacerbated by hyperglycemia [4]. These conditions create an environment conducive to microbial colonization and infection, often involving polymicrobial communities of aerobic and anaerobic organisms. Among the frequently implicated aerobic pathogens are *Staphylococcus aureus*, *Escherichia coli*, *Klebsiella pneumoniae*, *Pseudomonas aeruginosa*, and *Acinetobacter baumannii* [5]. The management of DFUs is further complicated by infections caused by multidrug-resistant (MDR) bacteria, which are increasingly prevalent in chronic wounds [6]. The global crisis of antimicrobial resistance (AMR), exacerbated by the misuse of antibiotics, has severely limited therapeutic options, particularly in infections involving DFUs [7,8].

The clinical challenges posed by DFUs are magnified by the frequent isolation of MDR pathogens, such as methicillin-resistant *Staphylococcus aureus* (MRSA) and carbapenem-resistant Gram-negative bacteria. MRSA complicates treatment by exhibiting resistance to methicillin and other β-lactam antibiotics, often necessitating the use of alternatives like vancomycin or linezolid [9,10]. Similarly, Gram-negative bacteria, including *Escherichia coli* and *Klebsiella pneumoniae*, are often producers of extended-spectrum β-lactamases (ESBLs) and carbapenemases, rendering them resistant to multiple antibiotic classes [11]. Carbapenem-resistant strains, particularly *Pseudomonas aeruginosa* and *Acinetobacter baumannii*, pose significant treatment challenges due to their intrinsic resistance mechanisms, ability to acquire additional resistance genes, and biofilm formation, which impairs immune clearance and antibiotic efficacy [12].

Carbapenemase-producing organisms, such as *Klebsiella pneumoniae* carbapenemase (KPC) and New Delhi metallo-β-lactamase (NDM) producers, represent a critical threat in DFU management. These enzymes confer resistance to carbapenems, a cornerstone of treatment for severe Gram-negative infections, and are often encoded on mobile genetic elements that facilitate their rapid dissemination [13,14,15]. The rise of these pathogens has been linked to poor clinical outcomes, including persistent infections, prolonged hospital stays, and higher amputation rates [16,17]. Resistance mechanisms in these bacteria include the production of carbapenemases, efflux pumps, porin modifications, and altered drug targets, significantly limiting therapeutic options [8,18].

This study aimed to evaluate the burden of MDR bacteria in DFUs, with a particular focus on carbapenemase production. Through phenotypic assays, such as the modified Hodge test and Carba NP test, alongside molecular techniques like PCR for genes encoding carbapenemases (e.g., *blaKPC*, *blaNDM*, *blaVIM*, *blaIMP*, and *blaOXA-48*), we sought to elucidate the resistance mechanisms and their correlation with clinical outcomes. The findings will contribute to developing targeted infection control strategies and promoting antibiotic stewardship programs. Additionally, the study highlights the urgent need for innovative therapeutic approaches, including combination therapies, antimicrobial peptides, and bacteriophage-based treatments, to address the growing challenge of MDR infections in DFUs.

## 2. Materials and Methods

### 2.1. Study Design and Setting

This 36-month retrospective, observational study was conducted from January 2021 until December 2023 at the Diabetic Foot Care Clinic (DFCC) and Microbiology Department of a tertiary care center. The study protocol received approval from the Institutional Ethics Committee of the Hail Health Cluster (Approval No: H-08-L-074) and the University of Hail (H-2023-377), Saudi Arabia.

### 2.2. Study Population

**Figure 1:**
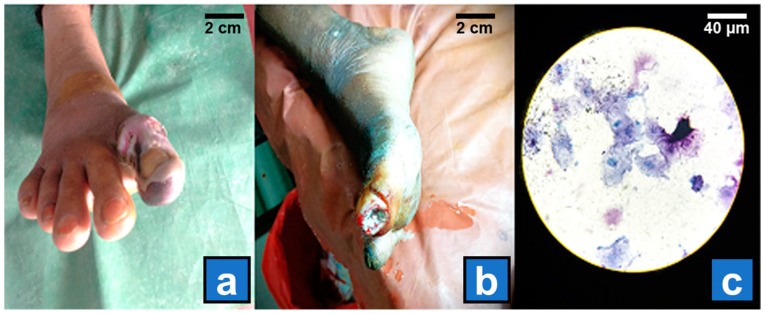
(**a**) Grade 3 diabetic foot ulcer from where biopsy samples were collected. (**b**) Grade 2 ulcer from which swab samples were collected for microbiological processing. (**c**) An image of Gram staining prepared from the swab sample shows some GPC in pairs.

A cohort of 200 patients with clinically confirmed DFUs from the clinician was included in this study. The inclusion criteria required participants to be 18 years or older, be diagnosed with type 1 or type 2 diabetes mellitus, and present with a foot ulcer classified as grade 2 or higher per the Wagner–Meggitt system (Figure 1a,b). The exclusion criteria included patients with active malignancy, those receiving immunosuppressive therapy, or those who had received antibiotic treatment within two weeks before coming to the DFCC.

### 2.3. Clinical Assessment and Ulcer Grading

In this retrospective observational study, clinical data for each patient were extracted from the Diabetic Foot Care Center (DFCC) records. The data included detailed clinical evaluations such as medical history, glycemic control (assessed by HbA1c levels), and complications like peripheral neuropathy and peripheral arterial disease. Ulcers were graded using the Wagner–Meggitt classification system, which evaluates ulcer depth, the extent of necrosis, and bone involvement [19].

The samples analyzed in this study were collected from ulcers that were classified as infected according to the Infectious Diseases Society of America (IDSA) guidelines [20]. Ulcers were considered infected if at least two of the following signs of infection were documented: erythema, warmth, swelling, pain, purulent discharge, or foul odor.

Ulcer swabs and tissue biopsies were obtained directly from the ulcer bed and placed immediately into Robertson’s Cooked Meat (RCM) medium and transported to the microbiology laboratory within 30 min to maintain sample integrity, following standard protocols [21].

### 2.4. Microbiological Processing

The swab and tissue samples were cultured within two hours of collection. For aerobic cultures, swabs were inoculated onto 5% sheep blood agar, MacConkey agar, and chocolate agar plates, which were incubated at 37 °C in ambient air and 5% CO_2_ for 24–48 h [22]. Bacterial growth was recorded, and colonies were identified via Gram staining (Figure 1c) and standard biochemical tests. Anaerobic cultures were performed by inoculating samples onto pre-reduced anaerobic agar (PRAS) media and incubating them at 37 °C in an anaerobic chamber for 48–72 h. The identification of anaerobes was performed via Gram staining and biochemical reactions.

Antimicrobial susceptibility testing (AST) was conducted via the Kirby–Bauer disk diffusion method on Mueller–Hinton agar, following CLSI guidelines [23]. The minimum inhibitory concentrations of antibiotics such as vancomycin, linezolid, and colistin were determined for MDR isolates via the E-test method [24]. The tested antibiotics included amoxicillin-clavulanate, piperacillin-tazobactam, ceftazidime, ceftriaxone, ciprofloxacin, gentamicin, imipenem, and tigecycline [25]. In addition, the isolates were screened for biofilm production via the microtiter plate method, with biofilm formation categorized as strong, moderate, weak, or nonproducer based on optical density readings.

### 2.5. Molecular Characterization of Resistance Mechanisms

For MDR isolates, molecular characterization of resistance genes was performed via PCR. The targeted resistance genes included mecA for MRSA; blaCTX-M, blaSHV, and blaTEM for ESBLs; vanA and vanB for vancomycin-resistant *Enterococcus faecium* (VRE); and blaKPC, blaNDM, and blaOXA-48 for carbapenemase production [26]. These gene targets were selected to identify key mechanisms of antibiotic resistance among MDR pathogens.

### 2.6. Modified Hodge Test (MHT)

MHT was employed for the phenotypic detection of carbapenemase activity in carbapenem-resistant isolates [27]. An *Escherichia coli* ATCC 25922 suspension was inoculated on a Mueller–Hinton agar plate, with a carbapenem disk placed centrally. Test organisms were streaked from the edge of the disk toward the periphery of the plate. Enhanced bacterial growth along the streak indicated carbapenemase production, confirming the presence of the enzyme in the resistant isolates [28].

### 2.7. Carba NP Test

Carbapenemase production was also confirmed via the Carba NP test, a rapid colorimetric assay based on the hydrolysis of carbapenem by carbapenemase enzymes [29]. Isolates that produce carbapenemase turn the solution from red to yellow, indicating positive results.

### 2.8. Molecular Detection of Carbapenemase Genes

All carbapenem-resistant isolates were subjected to molecular analysis to detect specific carbapenemase-encoding genes via PCR. The following genes were targeted: (i) blaKPC, (ii) blaNDM, (iii) blaVIM, (iv) blaOXA-48, and (v) blaIMP [30]. PCR was conducted via the following protocol. First, we performed DNA extraction via a standard boiling method. Second, we performed PCR amplification using specific primers for the abovementioned genes. Finally, the amplified products were run on a 1.5% agarose gel, stained with ethidium bromide, and visualized under UV light.

### 2.9. Statistical Analysis

The data were entered into a standardized Microsoft Excel 2019 database and analyzed via SPSS version 26.0 (IBM, Armonk, NY, USA). Descriptive statistics summarize demographic and clinical characteristics, with continuous variables reported as the means ± standard deviations (SDs) and categorical data reported as frequencies and percentages. The prevalence of MDR organisms in diabetic foot infections (DFIs) was calculated, and associations with clinical factors (e.g., ulcer grade, diabetes duration, and HbA1c) were evaluated via chi-square tests or Fisher’s exact tests. Multivariate logistic regression identified independent predictors of MDR infections, with statistical significance set at *p* < 0.05.

## 3. Results

The present study predominantly consisted of males, representing 60% of the participants, while females accounted for 40%. The mean age of the patients was 63.5 ± 10.5 years, with an average duration of type 2 diabetes mellitus (T2DM) of 8.6 ± 1.5 years. The average body mass index (BMI) was 24.9 ± 5.7 kg/m^2^, and 56.2% of the patients had a body weight between 51 and 75 kg. Notably, smoking was prevalent among 65.7% of participants.

Ulcer severity was classified using Wagner’s ulcer classification system, ranging from grades 2 to 5. A significant proportion of patients (67.5%) had experienced ulcers for more than one month before hospitalization. The average HbA1c level was 8.6 ± 1.5%, with 83.2% of patients demonstrating poor glycemic control (HbA1c > 7%) at admission. Furthermore, 75.5% of patients presented with pyrexia upon admission.

Comorbidities were common among the study population. Neuropathy was observed in 83%, retinopathy in 77.5%, hypertension in 54%, and nephropathy in 28.5% of patients. Regarding microbiological findings, 41.5% of infections were monomicrobial, while 58.5% were polymicrobial. Biofilm formation was detected in 90% of the isolates.

The study also revealed that 63% of ulcers were non-necrotic. Ulcers larger than 5 cm^2^ were present in 76.5% of patients, while the remaining 23.2% had ulcers smaller than 5 cm^2^. Infection types were categorized as superficial (40%), subcutaneous (30%), osteomyelitis (20%), and forefoot gangrene (10%)

Surgical intervention was necessary in 156 (78%) patients, with debridement in 83 (53.2%) being the most frequently performed procedure. Amputation was required in 73 (46.8%) cases, comprising 47 (64.4%) minor amputations and 26 (35.6%) major amputations. The mortality rate during hospitalization was nine (4.5%), and the mean hospital stay was 27.9 ± 6.2 days. For details, see Table 1.

(A) Study of association between demographical and clinical characteristics with MDR and Non-MDR bacterial isolates in DFU patients

The findings revealed significant associations between multidrug-resistant (MDR) infections and critical clinical parameters. MDR cases were strongly associated with pyrexia (88% vs. 71.3%, *p* = 0.017) and recent antibiotic use (58% vs. 34.7%, *p* = 0.003). Notably, minor amputations (76% vs. 6%), major amputations (38% vs. 4.7%), and polymicrobial infections (82% vs. 50.7%) demonstrated strong associations (*p* < 0.001). Wagner ulcer grade ≥II was more common in MDR cases (78% vs. 54%, *p* = 0.002). These results accentuate the link between MDR infections, advanced clinical presentations, polymicrobial involvement, and prior antibiotic exposure, highlighting the urgent need for robust antimicrobial stewardship and infection control measures. For details, refer to Table 2.

(B) Bacterial Diversity in DFU Infections

**Figure 2:**
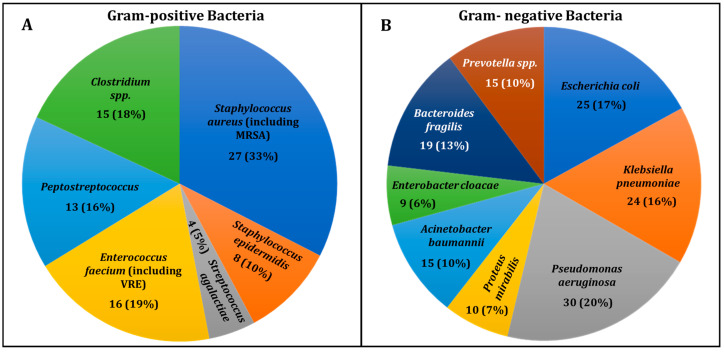
Distribution of Gram-positive (**A**) and Gram-negative (**B**) bacteria isolated from DFU infections. The chart depicts the prevalence of different bacterial groups in DFU samples, highlighting the predominant bacterial types involved in these infections.

The distribution of Gram-positive bacteria isolated from DFU samples is shown in Figure 2A. Staphylococcus aureus, including MRSA, was the most common, found in 27 cases (33%), consistent with its role in skin infections in diabetic patients. Other organisms included *Staphylococcus epidermidis* (8 cases, 10%); *Enterococcus faecium*, including VREs (16 cases, 19%); and *Streptococcus agalactiae* (4 cases, 5%). Anaerobic bacteria, such as *Peptostreptococcus* (13 cases, 16%) and *Clostridium* spp. (15 cases, 18%), were also identified, highlighting the polymicrobial nature of DFUs, especially in deep infections. This underscores the diversity of bacterial species in these complex infections.

Figure 2B illustrates the distribution of Gram-negative bacteria in DFU infections. The most prevalent were *Pseudomonas aeruginosa* (30 cases, 20%) and *Klebsiella pneumoniae* (24 cases, 16%), both commonly associated with chronic wounds and MDR profiles. *Escherichia coli* appeared in 25 cases (17%), reflecting its frequent occurrence in DFUs. Supplementary Table S1 provides details on microbiological analysis, MDR status, and antimicrobial resistance.

Other notable organisms included *Proteus mirabilis*, *Acinetobacter baumannii*, *Bacteroides fragilis*, *Enterobacter cloacae*, and *Prevotella* spp. Anaerobes like Bacteroides and Prevotella, thriving in chronic wound environments, further complicate treatment. The diversity of pathogens underscores the need for thorough microbiological analysis to inform effective therapy.

(C) Renal Status in Patients with Diabetic Foot Ulcer Infections

In the comprehensive dataset of 200 patients, renal complications were highly prevalent, with a significant proportion of individuals diagnosed with varying stages of chronic kidney disease (CKD). The majority of patients presented with CKD stages 3 and 4, with eGFR levels ranging from 15 to 59 mL/min/1.73 m^2^, indicating moderate to severe renal impairment. Notably, CKD stage 4 (eGFR: 15–29 mL/min/1.73 m^2^) was the most common stage, affecting nearly 40% of the cohort, and 15% of patients had progressed to CKD stage 5, characterized by end-stage renal disease (eGFR ≤ 15 mL/min/1.73 m^2^). These patients often exhibited significantly elevated serum creatinine levels, with values frequently exceeding 3.0 mg/dL, reflecting impaired kidney function. In contrast, a smaller proportion (approximately 25%) presented with CKD stage 2 (eGFR: 60–89 mL/min/1.73 m^2^), indicating mild renal dysfunction (Supplementary Table S1).

The renal dysfunction observed in these patients was exacerbated by the presence of multidrug-resistant bacterial infections, with *Klebsiella pneumoniae*, *Pseudomonas aeruginosa*, and *Acinetobacter baumannii* emerging as the predominant pathogens. The molecular characterization revealed high resistance levels, with multiple carbapenemase-producing strains, including *bla_NDM-1*, *bla_VIM*, *bla_KPC*, and *bla_OXA-48*, detected across various isolates. These resistant organisms contributed to more complicated clinical courses and were associated with poor outcomes, especially in patients with existing renal impairment. The frequent overlap of diabetes, chronic infection, and renal dysfunction highlights the urgent need for comprehensive management strategies, including tailored antimicrobial therapy, early detection of kidney involvement, and close monitoring of renal function in diabetic foot ulcer patients. The data reinforce the complex interplay between kidney disease and infection in this vulnerable patient population, necessitating a multidisciplinary approach to improve patient care and outcomes.

(D) Distribution of multidrug resistance genes in clinically significant bacterial pathogens

**Figure 3:**
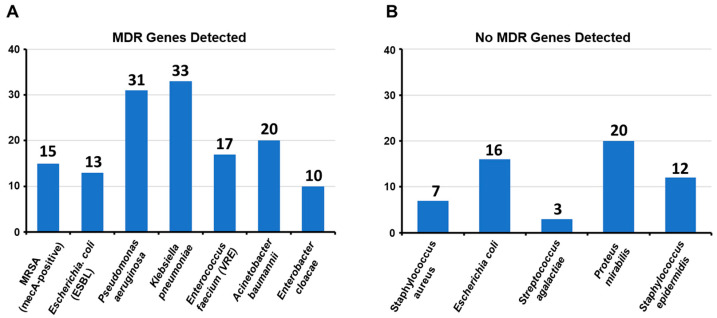
Presence (**A**) and absence (**B**) of MDR genes in clinically significant bacterial pathogens isolated from diabetic foot ulcer infections.

Figure 3A shows the detection of MDR genes in seven clinically significant bacterial pathogens, major contributors to healthcare-associated infections. *Klebsiella pneumoniae* (33 genes) and *Pseudomonas aeruginosa* (31 genes) exhibited the highest MDR gene counts, emphasizing their ability to resist multiple antibiotics and their role in severe hospital-acquired infections, including pneumonia and bloodstream infections. *Acinetobacter baumannii* (20 genes) and *Enterococcus faecium* (VRE, 17 genes) also showed notable resistance, complicating treatment, particularly for immunocompromised patients. These findings highlight the urgent need for strategies to address MDR in healthcare settings.

Other notable pathogens included MRSA (mecA-positive) with 15 MDR genes and *Escherichia coli* (ESBL) with 13 genes, both key contributors to hospital and community-associated infections. *Enterobacter cloacae* exhibited 10 MDR genes, reflecting a lower but still significant resistance level. The absence of MDR genes in the five bacterial species suggested that these pathogens lacked significant resistance mechanisms, as shown in Figure 3B.

*Proteus mirabilis* had the highest frequency (20 cases) where no MDR genes were detected, followed by *E. coli* (16 cases). These bacteria, often associated with urinary tract infections, may remain susceptible to standard antibiotic regimens. *Staphylococcus epidermidis* and *Staphylococcus aureus* were detected in moderate cases with no MDR genes, with 10 and 7 cases, respectively. This is particularly relevant for *Staphylococcus aureus*, a pathogen typically associated with methicillin resistance, suggesting that in these cases, it has not developed significant drug resistance. *Streptococcus agalactiae*, with four cases, was present in the lowest number, yet this organism is generally considered to be less prone to acquiring MDR genes (Supplementary Table S2).

These findings highlight that while these pathogens can still pose clinical challenges, the absence of MDR genes in these cases provides an opportunity for effective treatment with conventional antibiotics. However, continuous monitoring is essential to prevent the emergence of resistance in these bacteria.

(E) MHT, Carba NP Test, and Molecular Detection of Carbapenemase Genes from *E. coli* Isolates
  (i) Carbapenemase Production in *Escherichia coli* Isolates

Carbapenem resistance in *Escherichia coli* has become a significant public health concern, particularly in chronic infections such as DFUs. In this study, we evaluated 29 *E. coli* isolates from infected DFUs for carbapenemase production via a combination of phenotypic and molecular techniques (Table 3). The aim was to understand the prevalence and distribution of key carbapenemase enzymes and their associated genes, specifically *blaKPC*, *blaNDM*, *blaVIM*, *blaIMP*, and *blaOXA-48*.

 (ii) Phenotypic and Molecular Detection of Carbapenemase Production and Prevalence of Carbapenemase Genes

The MHT and Carba NP tests were employed to phenotypically detect carbapenemase production in the isolates. MHT identifies carbapenemase production through enhanced growth of a carbapenem-susceptible strain in the presence of a carbapenemase producer, while the Carba NP test rapidly detects carbapenemase activity via colorimetric changes caused by carbapenem hydrolysis. Both tests are crucial for identifying carbapenemase producers, which exhibit resistance to nearly all beta-lactam antibiotics, including last-resort drugs such as imipenem, meropenem, and ertapenem.

Among 29 isolates, 21 (72.4%) were positive for carbapenemase production based on MHT and Carba NP results, confirming the presence of carbapenemase enzymes. The remaining eight isolates (27.6%) were phenotypically negative, suggesting alternative resistance mechanisms such as porin loss or efflux pumps. PCR analysis of all isolates targeted clinically significant carbapenemase genes (*blaKPC*, *blaNDM*, *blaVIM*, *blaIMP*, and *blaOXA-48*). Of the phenotypically positive isolates, eleven (52.4%) carried *blaNDM*, and ten (47.6%) carried *blaKPC*, while no isolates tested positive for *blaVIM*, *blaIMP*, or *blaOXA-48*.

The detection of *blaNDM* highlights its role as a significant carbapenemase mechanism. *NDM* is a potent enzyme capable of hydrolyzing a broad range of beta-lactams and is often plasmid-mediated, facilitating its spread across bacterial species and geographic regions. Similarly, the detection of *blaKPC* underscores its clinical relevance due to its ability to confer resistance to all beta-lactams. While *blaKPC* was initially identified in *Klebsiella pneumoniae*, its presence in *E. coli* from DFU is concerning given the chronic nature of these infections and their association with poor outcomes.

The absence of *blaVIM*, *blaIMP*, and *blaOXA-48* suggests that NDM and KPC are the dominant carbapenemase mechanisms in this cohort of *E. coli* isolates from DFUs, reflecting local epidemiological trends. These findings emphasize the importance of early detection and infection control measures to prevent the spread of carbapenemase producers in clinical settings.

(F) MHT, Carba NP Test, and Molecular Detection of Carbapenemase Genes of *Pseudomonas aeruginosa* Isolates

Table 4 summarizes the carbapenemase production and genetic characterization of 31 *Pseudomonas aeruginosa* isolates, with a focus on phenotypic testing via the MHT and Carba NP tests, as well as the molecular detection of key carbapenemase genes (*blaKPC*, *blaNDM*, *blaVIM*, *blaIMP*, and *blaOXA-48*). Each isolate was tested for resistance to imipenem and meropenem, which are among the primary carbapenems used to treat infections caused by *Pseudomonas aeruginosa*. Ertapenem testing was omitted as this antibiotic is not effective against *Pseudomonas aeruginosa* due to intrinsic resistance.

 (i) Phenotypic detection of carbapenemase production

In Table 4, a positive MHT result is indicated by enhanced bacterial growth around the carbapenem disk, confirming carbapenemase production. Among the 31 isolates, 23 (74.2%) tested positive for carbapenemase production via the MHT. The remaining nine isolates (29%) tested negative for carbapenemase production in this phenotypic assay, indicating the absence of detectable carbapenemase activity via this method. In all cases, the results of the Carba NP test were consistent with those of the MHT, further confirming the presence of carbapenemase enzymes in 23 isolates (74.2%), whereas the remaining eight isolates were negative.

 (ii) Carbapenemase Genes Detected by PCR and Their Correlation with Antibiotic Susceptibility to Imipenem and Meropenem

Following phenotypic tests, PCR was performed to detect *blaKPC*, *blaNDM*, *blaVIM*, *blaIMP*, and *blaOXA-48*, the most common carbapenemase genes in *Pseudomonas aeruginosa* and other Gram-negative bacteria. Among phenotypically positive (23/31) isolates, seven (30.4%) carried the *blaNDM* gene, a metallo-β-lactamase that confers high-level resistance to nearly all β-lactam antibiotics. The presence of *NDM* is concerning due to its plasmid-mediated transmission, facilitating the rapid spread of resistance. Examples of *blaNDM*-positive isolates (e.g., 1, 7, and 11) were confirmed via MHT and Carba NP tests.

Eight isolates (34.8%) were positive for the *blaVIM* gene, another metallo-β-lactamase capable of hydrolyzing carbapenems and other β-lactams. These isolates (e.g., 2, 8, 12) also showed concordant results in phenotypic tests. Additionally, seven isolates (30.4%) carried the *blaKPC* gene, a serine β-lactamase that hydrolyzes a broad range of β-lactams. *KPC*, initially identified in *Klebsiella pneumoniae*, poses a serious challenge when detected in *P. aeruginosa*. Examples include isolates 5, 9, and 14, which tested positive in phenotypic assays.

None of the isolates carried *blaIMP* or *blaOXA-48*, enzymes more commonly associated with other Gram-negative species, indicating that resistance in this cohort was predominantly mediated by *NDM*, *VIM*, and *KPC*. The zone diameters for imipenem and meropenem, measured to assess susceptibility, ranged from 14 to 24 mm and 12 to 21 mm, respectively. Isolates with smaller diameters (e.g., imipenem: 14 mm, isolate 5) demonstrated significant resistance, consistent with carbapenemase production. In contrast, isolates with larger diameters (e.g., meropenem: 18 mm, isolate 13) lacked carbapenemase genes, suggesting alternative resistance mechanisms or susceptibility.

(G) MHT, Carba NP Test, and Molecular Detection of Carbapenemase Genes of *Klebsiella pneumoniae* Isolates

Table 5 presents the results of carbapenemase production and genetic characterization for 33 *Klebsiella pneumoniae* isolates from DFU. The key parameters include the results of phenotypic testing via MHT and the Carba NP test, alongside the molecular detection of five important carbapenemase genes: *blaKPC*, *blaNDM*, *blaVIM*, *blaIMP*, and *blaOXA-48*. Each isolate’s susceptibility to carbapenems, including imipenem, meropenem, and ertapenem, is reported via zone diameters (in millimeters), which are used to indicate resistance or susceptibility to these antibiotics.

 (i) Phenotypic and Molecular Detection of Carbapenemase Production and Genes in *Klebsiella pneumoniae* isolates

In Table 5, 24 of the 33 isolates (72.7%) tested positive for carbapenemase production using the MHT, confirming resistance to at least one carbapenem antibiotic. All MHT-positive isolates were also confirmed by the Carba NP test, providing strong evidence of carbapenemase production. The remaining nine isolates (24.2%) were negative in both tests. To identify the genetic basis of resistance, PCR was conducted to detect *blaKPC*, *blaNDM*, *blaVIM*, *blaIMP*, and *blaOXA-48*.

Fourteen isolates (58.3%) carried the *blaKPC* gene, encoding a serine-based β-lactamase responsible for hydrolyzing carbapenems, cephalosporins, and penicillins. Isolates such as 11, 14, and 17 showed high carbapenem resistance with small zone diameters for imipenem, meropenem, and ertapenem. Eight isolates (33.3%) were positive for the *blaNDM* gene, encoding a metallo-β-lactamase that confers resistance to nearly all β-lactams. Examples include isolates 2, 12, and 15, which were resistant to all carbapenems tested. Two isolates (8.3%) carried the *blaVIM* gene, encoding Verona integron-encoded metallo-β-lactamase, detected in isolates 8 and 18.

No isolates tested positive for *blaIMP* or *blaOXA-48*. These genes, typically associated with pathogens like *Acinetobacter baumannii* and *Pseudomonas aeruginosa*, were absent in the *Klebsiella pneumoniae* isolates studied.

 (ii) Antibiotic Susceptibility to Carbapenems in Non-Carbapenemase-Producing Isolates

Table 5 reports zone diameters for imipenem, meropenem, and ertapenem, with smaller diameters indicating higher carbapenem resistance based on CLSI breakpoints (≤19 mm for imipenem/meropenem and ≤18 mm for ertapenem). Zone diameters ranged from 9 mm to 22 mm. The most resistant isolates, such as 9 and 19, had small diameters (≤15 mm) and were typically positive for *blaKPC* or *blaNDM*. Meropenem resistance was reflected by diameters as low as 11 mm, with highly resistant isolates (e.g., 9, 19, and 24) confirmed as carbapenemase producers. Ertapenem, a key indicator of carbapenemase activity in *Klebsiella pneumoniae*, showed the smallest zone diameters, with values as low as 9 mm in resistant isolates.

Notably, nine isolates (27.3%) were negative for carbapenemase production in both phenotypic and molecular tests. These isolates (e.g., 3, 16, and 27) exhibited larger zone diameters, indicating intermediate resistance or susceptibility. The absence of carbapenemase genes in these isolates suggested alternative mechanisms, such as porin loss or efflux pump activity, contributed to their reduced carbapenem susceptibility.

(H) MHT, Carba NP test, and molecular detection of carbapenemase genes of *Acinetobacter baumannii* isolates

Table 6 summarizes the carbapenemase production and genetic characterization of 20 *Acinetobacter baumannii* clinical isolates from DFU. The dataset includes phenotypic results from the MHT and the Carba NP test, alongside molecular detection of the primary carbapenemase-encoding genes (*blaKPC*, *blaNDM*, *blaVIM*, *blaIMP*, and *blaOXA-48*). Table 5 also lists the zone diameters for imipenem, meropenem, and ertapenem, providing insight into each isolate’s level of carbapenem resistance.

 (i) Phenotypic and Molecular Detection of Carbapenemase Production and Genes in *Acinetobacter baumannii* isolates

In this study, MHT and Carba NP tests were used to detect carbapenemase production in *Acinetobacter baumannii* isolates. Of the 20 isolates, 14 (70%) were positive for carbapenemase production by MHT, confirming that carbapenemase enzymes were primarily responsible for carbapenem resistance. All MHT-positive isolates were also confirmed by the Carba NP test. PCR testing for carbapenemase genes, including *blaKPC*, *blaNDM*, *blaVIM*, *blaIMP*, and *blaOXA-48*, revealed that four isolates (26.7%) carried the *blaKPC* gene, showing resistance to all tested carbapenems with small zone diameters (≤18 mm). Four isolates (26.7%) were positive for *blaNDM*, with high resistance to imipenem and meropenem (zone diameters ≤ 14 mm). Seven isolates (46.7%) carried the *blaIMP* gene, showing significant resistance with small zone diameters for all carbapenems. However, five isolates (25%) were negative for all tested carbapenemase genes, indicating other resistance mechanisms, such as efflux pumps or porin loss, despite their reduced carbapenem susceptibility (zone diameters ≤ 20 mm). These findings highlight the prevalence of carbapenemase production in *A. baumannii* and the presence of alternative resistance mechanisms in some isolates.

 (ii) Antibiotic Susceptibility and Resistance Mechanisms in Non-Carbapenemase Producers

Table 6 summarizes the zone diameters for imipenem, meropenem, and ertapenem, revealing resistance patterns in each isolate based on CLSI guidelines (resistance: ≤19 mm for imipenem and meropenem; ≤18 mm for ertapenem). Carbapenemase-producing isolates, such as isolate 1 (imipenem 15 mm), showed high resistance with zone diameters below the cutoff. Non-carbapenemase producers (e.g., isolates 5, 10, 15, and 17) exhibited larger diameters (≥19 mm), indicating intermediate or reduced resistance. Meropenem results followed a similar trend, with diameters ranging from 9 mm to 19 mm; carbapenemase producers, like isolate 3 (15 mm, KPC-positive), had smaller diameters, consistent with high resistance. Although *Acinetobacter baumannii* is intrinsically resistant to ertapenem, all isolates showed resistance (7–18 mm). Carbapenemase producers, such as isolate 4 (ertapenem 9 mm), displayed the smallest diameters.

Notably, five isolates (25%) were negative for carbapenemase genes but still showed reduced susceptibility, suggesting alternative mechanisms like efflux pumps, porin mutations, or other β-lactamases. Further studies are necessary to clarify these resistance pathways.

(I) MHT, Carba NP Test, and Molecular Detection of Carbapenemase Genes of *Enterobacter cloacae* isolates

Table 7 summarizes the carbapenemase production and genetic characterization of 10 *Enterobacter cloacae* isolates from DFU. It includes results from phenotypic assays such as MHT and the Carba NP Test, along with molecular detection of key carbapenemase-encoding genes such as *blaKPC*, *blaNDM*, *blaVIM*, *blaIMP*, and *blaOXA-48*. The table also provides zone diameters for imipenem, meropenem, and ertapenem, three critical carbapenem antibiotics used to assess resistance levels.

 (i) Phenotypic and Molecular Detection of Carbapenemase Production and Antibiotic Susceptibility to Carbapenems

Table 7 highlights that eight isolates (80%) tested positive for carbapenemase production via MHT, confirmed by Carba NP, indicating carbapenemase enzymes as a key resistance mechanism in *Enterobacter cloacae*. PCR analysis detected diverse carbapenemase genes, including *blaKPC* (isolates 1 and 6, 25%), *blaNDM* (isolates 2, 7, and 10, 37.5%), *blaVIM* (isolate 3, 12.5%), and *blaIMP* (isolates 4 and 9, 25%). These isolates showed high resistance, with small zone diameters for imipenem, meropenem, and ertapenem (e.g., isolate 4: imipenem 12 mm, meropenem 10 mm, ertapenem 8 mm).

Two isolates (20%), negative for carbapenemase genes, also tested negative via MHT and Carba NP. Despite this, they exhibited intermediate resistance, possibly due to alternative mechanisms such as efflux pumps or porin mutations (e.g., isolate 5: imipenem 21 mm, meropenem 19 mm, ertapenem 17 mm). Zone diameters across all isolates ranged from 12 mm to 21 mm for imipenem and 10 mm to 19 mm for meropenem. Carbapenemase-positive isolates consistently had smaller diameters (e.g., isolate 9: 11 mm), while non-carbapenemase producers showed larger diameters, indicating reduced resistance or intermediate susceptibility. Ertapenem further distinguished carbapenemase producers (e.g., isolate 2: 11 mm) from nonproducers (e.g., isolate 8: 17 mm), underscoring its value as a marker of resistance.

## 4. Discussion

The findings of this study highlight a significant association between MDR infections and various clinical parameters in DFUs. Notably, the higher prevalence of pyrexia in MDR cases (88% vs. 71.3%, *p* = 0.017) emphasizes the more severe and systemic nature of infections caused by MDR pathogens. Pyrexia often indicates a systemic inflammatory response, which may be exacerbated in MDR infections due to these pathogens’ heightened virulence and resistance mechanisms [31]. However, the literature on the relationship between pyrexia and MDR in DFU remains limited. The systemic involvement underscores the aggressive nature of these infections, often necessitating prolonged hospitalization and intensive management [31].

The association between recent antibiotic use and MDR infections (58% vs. 34.7%, *p* = 0.003) highlights a concerning trend in antimicrobial misuse, which has been documented in a study from China [6]. Overuse and inappropriate prescription of antibiotics in DFIs contribute significantly to the emergence of resistant strains, particularly *Escherichia coli*, *Staphylococcus aureus*, *Pseudomonas aeruginosa*, and *Klebsiella pneumoniae*. These organisms are common culprits in DFUs and pose treatment challenges due to their resistance mechanisms [6].

The strong correlation between MDR infections and higher amputation rates (minor amputations 76% vs. 6%, major amputations 38% vs. 4.7%, *p* < 0.001) is of critical concern. Previous studies in Jeddah (Saudi Arabia) and India have reported similar findings, where patients with MDR infections were significantly more likely to undergo amputations due to poor wound healing and progressive tissue damage [32,33]. MDR pathogens delay effective treatment, leading to persistent infection, sepsis, and ultimately, surgical intervention as a last resort [32,33].

The study also highlights the high prevalence of polymicrobial infections in MDR cases (82% vs. 50.7%, *p* < 0.001). Polymicrobial infections, often involving Gram-negative bacilli and Gram-positive cocci, have been reported as a frequent finding in DFUs [32,34]. These infections complicate therapeutic management due to the need for broad-spectrum antibiotics and targeted treatment strategies. Moreover, polymicrobial infections often correlate with longer hospital stays, increased costs, and higher morbidity [32,34].

The association between MDR infections and advanced ulcer grades (Wagner grade ≤ II in 78% vs. 54%, *p* = 0.002) suggests that MDR infections are more prevalent in severe and chronic ulcers. Advanced grades, particularly Wagner III and above, are characterized by deeper tissue involvement, osteomyelitis, and increased bacterial load, which predispose ulcers to MDR pathogens. Similar trends have been reported in studies, where the severity of DFUs was a significant predictor of MDR infections [6,35].

The results of this study underscore the alarming prevalence of carbapenemase-producing MDR bacteria in DFU infections, a finding consistent with global and regional trends. Among the 200 clinical isolates analyzed, more than half were confirmed to produce carbapenemases, with *Klebsiella pneumoniae*, *Pseudomonas aeruginosa*, *Acinetobacter baumannii*, and *Escherichia coli* identified as the dominant pathogens. The detection of key resistance genes, including *blaKPC* and *blaNDM*, highlights the critical role of molecular mechanisms in driving antibiotic resistance in these pathogens.

Studies from Saudi Arabia have documented the increasing prevalence of carbapenem-resistant organisms in both healthcare and community settings. For instance, Ibrahim et al. (2019) reported the predominance of *blaNDM* and *blaOXA-48* genes among carbapenem-resistant Gram-negative bacteria isolated from clinical samples [36]. In addition, studies from Iraq have highlighted the rapid detection of *bla OXA*, *bla KPC*, and *bla NDM* among carbapenem-resistant *Klebsiella pneumoniae* isolated from various hospitals in Duhok City [37]. Our findings revealed a significant association between strong biofilm production and MDR status, with 60% of carbapenemase-producing isolates forming robust biofilms. Biofilms are known to protect bacteria from antibiotics and immune responses, thereby complicating treatment. Similar observations have been reported in Saudi-based studies, where biofilm-forming *Pseudomonas aeruginosa* and *Acinetobacter baumannii* were implicated in chronic wound infections [36]. The ability of these pathogens to persist in biofilm form further underscores the challenge of managing DFU infections caused by MDR bacteria.

While our study did not detect *blaVIM*, *blaIMP*, or *blaOXA-48* in the analyzed isolates, these genes have been reported in neighboring Gulf countries. A surveillance study from Kuwait identified *blaVIM* and *blaIMP* as prevalent carbapenemase genes in *Pseudomonas aeruginosa*, whereas *blaOXA-48* was more commonly associated with *Enterobacteriaceae* in Iran [38]. The absence of these genes in our isolates may reflect localized epidemiological differences, emphasizing the need for regional molecular surveillance to monitor resistance trends. Patients with carbapenemase-producing MDR infections in this study exhibited poorer clinical outcomes, including higher HbA1c levels (mean 9.1%) and more severe ulcers. This finding is consistent with reports from Saudi Arabia and the Middle East, where uncontrolled diabetes has been identified as a significant risk factor for MDR infections in DFUs. Haji et al. (2021) reported that patients with poorly controlled diabetes were more likely to develop infections caused by carbapenem-resistant organisms, highlighting the importance of glycemic control in reducing infection severity [39].

The high prevalence of carbapenemase-producing organisms in this study underscores the urgent need for robust antibiotic stewardship programs. Regional studies have emphasized the role of antimicrobial misuse in driving resistance, particularly in the Middle East, where over-the-counter availability of antibiotics remains a challenge. Effective stewardship requires the routine screening of carbapenemase genes, particularly *blaNDM* and *blaKPC*, to guide targeted therapy and prevent the unnecessary use of broad-spectrum antibiotics [40].

The limited treatment options for carbapenem-resistant infections necessitate the exploration of alternative therapies. Recent studies have highlighted the potential of antimicrobial peptides, bacteriophage therapy, and combination regimens as promising approaches to combat MDR bacteria. For example, phage therapy targeting *Pseudomonas aeruginosa* biofilms has shown efficacy in preclinical models, offering hope for managing biofilm-associated infections in DFUs [41].

Our findings contribute to the growing body of evidence on the molecular epidemiology of carbapenem resistance in DFUs. The predominance of *blaKPC* and *blaNDM* genes reflects global trends but also highlights regional nuances in resistance mechanisms. Further studies are needed to elucidate the role of mobile genetic elements, such as plasmids and transposons, in the dissemination of these genes across bacterial populations.

One of the key limitations of this study is its single-center design, which may restrict the generalizability of the findings to broader populations across different regions of Saudi Arabia and the Middle East. The relatively small sample size (200 isolates) could limit the detection of less prevalent resistance mechanisms or rare carbapenemase genes, such as blaIMP or blaOXA-48, which have been reported in other studies in the region. Additionally, the study focused on phenotypic and molecular detection of selected resistance genes but did not include whole-genome sequencing, which could provide a more comprehensive understanding of the genetic diversity and mobile genetic elements driving resistance. The absence of a longitudinal component also limits the ability to assess temporal trends in resistance patterns or the impact of specific interventions. Finally, clinical outcomes were not extensively correlated with microbiological findings, which could offer deeper insights into the implications of carbapenemase production on patient prognosis and treatment efficacy. Addressing these limitations in future research could strengthen the understanding of multidrug resistance in diabetic foot infections and guide more effective interventions.

## 5. Conclusions

The study revealed a high prevalence of carbapenemase-producing multidrug-resistant bacteria, particularly *Klebsiella pneumoniae, Pseudomonas aeruginosa, Acinetobacter baumannii,* and *Escherichia coli*, in diabetic foot ulcer infections, with the *blaKPC* and *blaNDM* genes being predominant. High rates of biofilm formation by these organisms complicate treatment by reducing antibiotic efficacy and facilitating the spread of resistance genes. The limited treatment options underscore the need for routine molecular screening of carbapenemase genes in DFUs to guide targeted therapy and improve antibiotic stewardship. Alternative therapies, including combination therapy, antimicrobial peptides, and bacteriophage therapy, may offer promising solutions for these challenging infections. Early intervention and strict infection control measures are critical to prevent the spread of MDR pathogens in DFU care settings.

## Figures and Tables

**Table 1:** Demographic and clinical characteristics of patients with DFU.

	Characteristics	Number of Isolates (%)
Sex	Male	120 (60%)
	Female	80 (40%)
	Age (years)	63.5 ± 10.5
	Body mass index (BMI) Kg/m^2^	24.9 ± 5.7
	Duration of diabetes (years)	8.6 ± 1.5
	HbA1c %	8.6 ± 1.5
Amputation	Minor	47 (64.4%)
	Major	26 (35.6%)
	Mortality	9 (4.5%)
Wagner ulcer grade	Grade II	80 (40%)
	Grade III	60 (30%)
	Grade IV	40 (20%)
	Grade V	20 (10%)
Ulcer size (cm^2^)	<5 cm^2^	47 (23.5%)
	>5 cm^2^	153 (76.5%)
Duration of ulcers	<1months	65 (32.5%)
	>1 months	135 (67.5%)
Clinical complications	Neuropathy	166 (83%)
	Retinopathy	155 (77.5%)
	Nephropathy	57 (28.5%)
	Hypertension	108 (54%)
	CVD	43 (21.5%)
Type of infection	Monomicrobial infection	83 (41.5%)
	Polymicrobial infection	117 (58.5%)
Biofilm production	Strong	50 (25%)
	Moderate	80 (40%)
	Weak	50 (25%)
	None	20 (10%)
Antibiotics used in last 1 month	Yes	81 (40.5%)
	No	119 (59.5%)
Nature of ulcer	Necrotic	74 (37%)
	Non-necrotic	126 (63%)
Sample type	Wound swab	128 (64%)
	Tissue biopsy	72 (36%)
Pyrexia	Yes	151 (75.5%)
	No	49 (24.5%)
Hospital stays (in days)		27.9 ± 6.2

Value is expressed as *n* (%) and mean ± SD.

**Table 2:** Patient characteristics and their association with MDR and non-MDR bacterial isolates in diabetic foot infection.

	Characteristics	MDR *n* (%)	Non-MDR *n* (%)	*p*-Value
Sex	Male	35 (70%)	85 (56.7%)	0.095
	Female	15 (30%)	65 (43.3%)	
Age (years)	<40	10 (20%)	26 (17.3%)	0.670
	>40	40 (80%)	124 (82.7%)	
Duration of diabetes (years)	<2	9 (18%)	31 (20.7%)	0.683
	>2	41 (82%)	119 (79.3%)	
Body mass index (kg/m^2^)	<16.7	6 (12%)	13 (8.7%)	0.488
	>16.7	44 (88%)	137 (91.3%)	
HbA1c (%)	<7	4 (8%)	17 (11.3%)	0.505
	>7	46 (92%)	133 (88.7%)	
Wound size (cm^2^)	<5	11 (22%)	36 (24%)	0.772
	>5	39 (78%)	114 (76%)	
Pyrexia	Yes	44 (88%)	107 (71.3%)	0.017 **
	No	6 (12%)	43 (28.7%)	
Minor amputation	Yes	38 (76%)	9 (6%)	<0.001 **
	No	12 (24%)	141 (94%)	
Major amputation	Yes	19 (38%)	7 (4.7%)	<0.001 **
	No	31 (62%)	143 (95.3%)	
Wagner ulcer grade	<II	11 (22%)	69 (46%)	0.002 **
	>II	39 (78%)	81 (54%)	
Duration of ulcers (months)	<1	13 (26%)	52 (48.9%)	0.257
	>1	37 (74%)	98 (65.3%)	
Sample type	Wound swab	34 (68%)	94 (62.7%)	0.496
	Tissue biopsy	16 (32%)	56 (37.3%)	
Type of infection	Polymicrobial	41 (82%)	76 (50.7%)	<0.001 **
	Monomicrobial	9 (18%)	74 (49.3%)	
Nature of ulcer	Necrotic	14 (28%)	60 (40%)	0.127
	Non-necrotic	36 (72%)	90 (60%)	
Biofilm	Biofilm producer	42 (84%)	138 (92%)	0.102
	Biofilm non-producer	8 (16%)	12 (8%)	
Antibiotic used in last 1 month	Yes	29 (58%)	52 (34.7%)	0.003 **
	No	21 (42%)	98 (65.3%)	

Value is expressed as *n* (%). ** Denotes *p* < 0.05, i.e., statistically significant.

**Table 3:** Carbapenemase activity and genetic profiling of *E. coli* isolates from DFU.

Isolate No.	IMP (mm)	MRP (mm)	ETP (mm)	MHT	Carba NP Test	KPC	NDM	VIM	IMP	OXA-48	Carbapenemase Type
1	25	20	18	+	+	-	+	-	-	-	NDM
2	21	18	14	+	+	+	-	-	-	-	KPC
3	23	22	16	-	-	-	-	-	-	-	None
4	24	19	18	+	+	-	+	-	-	-	NDM
5	19	17	15	+	+	+	-	-	-	-	KPC
6	22	21	20	-	-	-	-	-	-	-	None
7	16	14	11	+	+	-	+	-	-	-	NDM
8	20	18	17	+	+	+	-	-	-	-	KPC
9	18	16	15	+	+	-	+	-	-	-	NDM
10	23	21	19	-	-	-	-	-	-	-	None
11	17	14	13	+	+	+	-	-	-	-	KPC
12	19	17	15	+	+	-	+	-	-	-	NDM
13	24	22	18	-	-	-	-	-	-	-	None
14	22	19	17	+	+	+	-	-	-	-	KPC
15	19	17	15	+	+	-	+	-	-	-	NDM
16	23	20	19	-	-	-	-	-	-	-	None
17	21	18	16	+	+	+	-	-	-	-	KPC
18	16	15	14	+	+	-	+	-	-	-	NDM
19	19	18	17	+	+	+	-	-	-	-	KPC
20	24	22	19	-	-	-	-	-	-	-	None
21	18	17	16	+	+	-	+	-	-	-	NDM
22	22	21	20	+	+	+	-	-	-	-	KPC
23	20	18	17	+	+	-	+	-	-	-	NDM
24	23	21	20	-	-	-	-	-	-	-	None
25	21	19	18	+	+	+	-	-	-	-	KPC
26	17	16	15	+	+	-	+	-	-	-	NDM
27	18	17	16	+	+	+	-	-	-	-	KPC
28	16	15	14	+	+	-	+	-	-	-	NDM
29	24	22	19	-	-	-	-	-	-	-	None

+ (positive); - (negative).

**Table 4:** Carbapenemase production and genetic characterization of *Pseudomonas aeruginosa* clinical isolates.

Isolate No.	IMP (mm)	MRP (mm)	ETP (mm)	MHT	Carba NP Test	KPC	NDM	VIM	IMP	OXA-48	Carbapenemase Type
1	20	18	N/A	+	+	-	+	-	-	-	NDM
2	16	14	N/A	+	+	-	-	+	-	-	VIM
3	22	19	N/A	-	-	-	-	-	-	-	None
4	18	17	N/A	+	+	-	-	-	-	-	IMP
5	15	12	N/A	+	+	+	-	-	-	-	KPC
6	24	20	N/A	-	-	-	-	-	-	-	None
7	17	15	N/A	+	+	-	+	-	-	-	NDM
8	19	16	N/A	+	+	-	-	+	-	-	VIM
9	14	12	N/A	+	+	+	-	-	-	-	KPC
10	12	20	N/A	-	-	-	-	-	-	-	None
11	18	15	N/A	+	+	-	+	-	-	-	NDM
12	16	14	N/A	+	+	-	-	+	-	-	VIM
13	21	18	N/A	-	-	-	-	-	-	-	None
14	14	22	N/A	+	+	+	-	-	-	-	KPC
15	20	16	N/A	+	+	-	-	+	-	-	VIM
16	15	12	N/A	+	+	-	+	-	-	-	NDM
17	24	21	N/A	-	-	-	-	-	-	-	None
18	19	17	N/A	+	+	-	-	+	-	-	VIM
19	14	13	N/A	+	+	+	-	-	-	-	KPC
20	17	14	N/A	+	+	-	+	-	-	-	NDM
21	15	12	N/A	+	+	-	-	+	-	-	VIM
22	18	16	N/A	-	-	-	-	-	-	-	None
23	16	14	N/A	+	+	-	+	-	-	-	NDM
24	20	17	N/A	+	+	+	-	-	-	-	KPC
25	14	12	N/A	+	+	-	-	+	-	-	VIM
26	22	19	N/A	-	-	-	-	-	-	-	None
27	17	15	N/A	+	+	+	-	-	-	-	KPC
28	18	16	N/A	+	+	-	+	-	-	-	NDM
29	21	18	N/A	-	-	-	-	-	-	-	None
30	19	17	N/A	+	+	+	-	-	-	-	KPC
31	14	13	N/A	+	+	-	-	+	-	-	VIM

N/A (Not applicable); + (positive); - (negative).

**Table 5:** Carbapenemase production and genetic characterization of *Klebsiella pneumoniae* clinical isolates from DFU.

Isolate No.	IMP (mm)	MRP (mm)	ETP (mm)	MHT	Carba NP Test	KPC	NDM	VIM	IMP	OXA-48	Carbapenemase Type
1	18	15	14	+	+	+	-	-	-	-	KPC
2	16	14	13	+	+	-	+	-	-	-	NDM
3	14	12	10	+	+	+	-	-	-	-	None
4	14	12	10	+	+	+	-	-	-	-	KPC
5	19	16	15	+	+	-	+	-	-	-	NDM
6	22	20	19	-	-	-	-	-	-	-	None
7	15	12	11	+	+	+	-	-	-	-	KPC
8	20	18	17	+	+	-	-	+	-	-	VIM
9	13	11	10	+	+	+	-	-	-	-	KPC
10	22	19	18	-	-	-	-	-	-	-	None
11	17	14	12	+	+	+	-	-	-	-	KPC
12	16	13	11	+	+	-	+	-	-	-	NDM
13	21	19	17	-	-	-	-	-	-	-	None
14	15	12	10	+	+	+	-	-	-	-	KPC
15	18	16	14	+	+	-	+	-	-	-	NDM
16	22	20	19	-	-	-	-	-	-	-	None
17	14	12	10	+	+	+	-	-	-	-	KPC
18	19	17	15	+	+	-	-	+	-	-	VIM
19	13	11	9	+	+	+	-	-	-	-	KPC
20	21	19	17	-	-	-	-	-	-	-	None
21	17	14	13	+	+	+	-	-	-	-	KPC
22	18	16	14	+	+	-	+	-	-	-	NDM
23	21	18	17	-	-	-	-	-	-	-	None
24	13	11	9	+	+	+	-	-	-	-	KPC
25	15	13	11	+	+	-	+	-	-	-	NDM
26	19	17	15	+	+	+	-	-	-	-	KPC
27	22	20	18	-	-	-	-	-	-	-	None
28	16	14	12	+	+	+	-	-	-	-	KPC
29	18	16	14	+	+	-	+	-	-	-	NDM
30	22	19	17	-	-	-	-	-	-	-	None
31	15	12	10	+	+	+	-	-	-	-	KPC
32	17	14	13	+	+	-	+	-	-	-	NDM
33	21	19	17	-	-	-	-	-	-	-	None

+ (positive); - (negative).

**Table 6:** Carbapenemase production and genetic characterization of *Acinetobacter baumannii* clinical isolates from DFU.

Isolate No.	IMP (mm)	MRP (mm)	ETP (mm)	MHT	Carba NP Test	KPC	NDM	VIM	IMP	OXA-48	Carbapenemase Type
1	15	12	10	+	+	-	-	-	+	-	IMP
2	14	13	11	+	+	-	+	-	-	-	NDM
3	17	15	12	+	+	+	-	-	-	-	KPC
4	13	11	9	+	+	-	-	-	+	-	IMP
5	20	18	17	-	-	-	-	-	-	-	None
6	14	12	10	+	+	-	-	-	+	-	IMP
7	16	14	11	+	+	-	+	-	-	-	NDM
8	18	16	15	+	+	+	-	-	-	-	KPC
9	12	10	8	+	+	-	-	-	+	-	IMP
10	22	19	18	-	-	-	-	-	-	-	None
11	13	11	9	+	+	-	+	-	-	-	NDM
12	12	13	10	+	+	-	-	-	+	-	IMP
13	18	16	14	+	+	+	-	-	-	-	KPC
14	12	10	8	+	+	-	-	-	+	-	IMP
15	20	18	17	-	-	-	-	-	-	-	None
16	16	12	10	+	+	-	+	-	-	-	NDM
17	19	17	16	-	-	-	-	-	-	-	None
18	16	13	12	+	+	+	-	-	-	-	KPC
19	11	9	7	+	+	-	-	-	+	-	IMP
20	22	19	18	-	-	-	-	-	-	-	None

+ (positive); - (negative).

**Table 7:** Carbapenemase production and genetic characterization of *Enterobacter cloacae* clinical isolates from DFU.

Isolate No.	IMP (mm)	MRP (mm)	ETP (mm)	MHT	Carba NP Test	KPC	NDM	VIM	IMP	OXA-48	Carbapenemase Type
1	16	14	13	+	+	+	-	-	-	-	KPC
2	15	13	11	+	+	-	+	-	-	-	NDM
3	19	17	16	+	+	-	-	+	-	-	VIM
4	12	10	8	+	+	-	-	-	+	-	IMP
5	21	19	17	-	-	-	-	-	-	-	None
6	15	13	11	+	+	+	-	-	-	-	KPC
7	18	16	14	+	+	-	+	-	-	-	NDM
8	20	18	17	-	-	-	-	-	-	-	None
9	13	11	10	+	+	-	-	-	+	-	IMP
10	14	12	11	+	+	-	+	-	-	-	NDM

+ (positive); - (negative).

## Data Availability

The data generated and analyzed during the current study are provided within the manuscript.

## Supplementary Materials

The following supporting information can be downloaded at: https://www.mdpi.com/article/10.3390/diagnostics15020141/s1, Table S1: Microbiological Analysis, MDR Status, and Antimicrobial Resistance in Patients with Diabetic Foot Ulcers; Table S2: Detection of MDR genes in clinical isolates.
