# Supplementary material for: Prevalence and Molecular Characterization of Carbapenemase-Producing Multidrug-Resistant Bacteria in Diabetic Foot Ulcer Infections
Source: Diagnostics (Basel). 2025 Jan 9;15(2):141. doi: 10.3390/diagnostics15020141 (PMC11763587; doi:10.3390/diagnostics15020141)
Supplement: Supplementary file 1 [file diagnostics-15-00141-s001.zip › diagnostics-3417603-supplementary.pdf]

Table S1: Microbiological Analysis, MDR Status, and Antimicrobial Resistance in Patients with Diabetic Foot Ulcers.

Patient ID	Sample Collection	Aerobic Culture	Anaerobic Culture	MDR Status	AST (Resistant)
P001	Swab	*Staphylococcus aureus*	None	No	None
P002	Biopsy	*Escherichia coli*	*Bacteroides fragilis*	Yes	Resistant: Cephalosporins, Fluoroquinolones
P003	Swab	*Pseudomonas aeruginosa*	None	Yes	Resistant: Carbapenems
P004	Biopsy	*Klebsiella pneumoniae*	*Peptostreptococcus*	Yes	Resistant: Aminoglycosides, Beta-lactams
P005	Swab	*Streptococcus agalactiae*	None	No	None
P006	Biopsy	*Enterococcus faecium*	*Clostridium perfringens*	Yes	Resistant: Vancomycin, Linezolid
P007	Swab	*Proteus mirabilis*	None	No	None
P008	Biopsy	*Acinetobacter baumannii*	*Prevotella spp.*	Yes	Resistant: Tigecycline, Colistin
P009	Swab	*Enterobacter cloacae*	None	Yes	Resistant: Cephalosporins, Carbapenems
P010	Biopsy	*Staphylococcus epidermidis*	*Bacteroides fragilis*	No	None
P011	Swab	*Staphylococcus aureus (MRSA)*	None	Yes	Resistant: Cephalosporins, Fluoroquinolones
P012	Biopsy	*Escherichia coli*	*Clostridium perfringens*	No	None
P013	Swab	*Proteus mirabilis*	None	No	None
P014	Biopsy	*Pseudomonas aeruginosa*	*Bacteroides fragilis*	Yes	Resistant: Carbapenems, Aminoglycosides
P015	Swab	*Enterococcus faecium (VRE)*	None	Yes	Resistant: Vancomycin, Linezolid
P016	Biopsy	*Klebsiella pneumoniae (ESBL)*	*Peptostreptococcus*	Yes	Resistant: Cephalosporins, Beta-lactams
P017	Swab	*Streptococcus agalactiae*	None	No	None
P018	Biopsy	*Acinetobacter baumannii*	*Prevotella spp.*	Yes	Resistant: Tigecycline, Colistin
P019	Swab	*Enterobacter cloacae*	None	Yes	Resistant: Carbapenems, Cephalosporins
P020	Biopsy	*Staphylococcus epidermidis*	*Bacteroides fragilis*	No	None
P021	Swab	*Escherichia coli (ESBL)*	None	Yes	Resistant: Cephalosporins, Fluoroquinolones
P022	Biopsy	*Pseudomonas aeruginosa*	*Clostridium spp.*	Yes	Resistant: Carbapenems, Aminoglycosides
P023	Swab	*Proteus mirabilis*	None	No	None

Patient ID	Sample Collection	Aerobic Culture	Anaerobic Culture	MDR Status	AST (Resistant)
P024	Biopsy	*Klebsiella pneumoniae*	*Bacteroides fragilis*	Yes	Resistant: Beta-lactams, Carbapenems
P025	Swab	*Staphylococcus aureus*	None	No	None
P026	Biopsy	*Acinetobacter baumannii*	*Peptostreptococcus*	Yes	Resistant: Colistin, Tigecycline
P027	Swab	*Enterococcus faecium (VRE)*	None	Yes	Resistant: Vancomycin, Linezolid
P028	Biopsy	*Escherichia coli*	*Clostridium spp.*	No	None
P029	Swab	*Pseudomonas aeruginosa*	None	Yes	Resistant: Carbapenems, Fluoroquinolones
P030	Biopsy	*Klebsiella pneumoniae (ESBL)*	*Prevotella spp.*	Yes	Resistant: Cephalosporins, Aminoglycosides
P031	Swab	*Escherichia coli*	None	No	None
P032	Biopsy	*Pseudomonas aeruginosa*	*Bacteroides fragilis*	Yes	Resistant: Carbapenems, Cephalosporins
P033	Swab	*Staphylococcus aureus (MRSA)*	None	Yes	Resistant: Cephalosporins, Fluoroquinolones
P034	Biopsy	*Enterobacter cloacae*	*Clostridium spp.*	Yes	Resistant: Carbapenems, Beta-lactams
P035	Swab	*Proteus mirabilis*	None	No	None
P036	Biopsy	*Klebsiella pneumoniae (ESBL)*	*Peptostreptococcus*	Yes	Resistant: Cephalosporins, Fluoroquinolones
P037	Swab	*Streptococcus agalactiae*	None	No	None
P038	Biopsy	*Acinetobacter baumannii*	*Prevotella spp.*	Yes	Resistant: Colistin, Tigecycline
P039	Swab	*Enterococcus faecium (VRE)*	None	Yes	Resistant: Vancomycin, Linezolid
P040	Biopsy	*Escherichia coli (ESBL)*	*Clostridium spp.*	Yes	Resistant: Cephalosporins, Carbapenems
P041	Swab	*Pseudomonas aeruginosa*	None	Yes	Resistant: Carbapenems, Aminoglycosides
P042	Biopsy	*Staphylococcus epidermidis*	*Bacteroides fragilis*	No	None
P043	Swab	*Klebsiella pneumoniae*	None	Yes	Resistant: Cephalosporins, Carbapenems
P044	Biopsy	*Acinetobacter baumannii*	*Peptostreptococcus*	Yes	Resistant: Tigecycline, Colistin
P045	Swab	*Proteus mirabilis*	None	No	None
P046	Biopsy	*Enterococcus faecium (VRE)*	*Prevotella spp.*	Yes	Resistant: Vancomycin, Linezolid
P047	Swab	*Escherichia coli*	None	No	None

Patient ID	Sample Collection	Aerobic Culture	Anaerobic Culture	MDR Status	AST (Resistant)
P048	Biopsy	*Staphylococcus aureus (MRSA)*	*Bacteroides fragilis*	Yes	Resistant: Cephalosporins, Fluoroquinolones
P049	Swab	*Pseudomonas aeruginosa*	None	Yes	Resistant: Carbapenems, Fluoroquinolones
P050	Biopsy	*Klebsiella pneumoniae (ESBL)*	*Peptostreptococcus*	Yes	Resistant: Cephalosporins, Beta-lactams
P051	Swab	*Staphylococcus aureus*	None	No	None
P052	Biopsy	*Escherichia coli (ESBL)*	*Clostridium spp.*	Yes	Resistant: Cephalosporins, Fluoroquinolones
P053	Swab	*Pseudomonas aeruginosa*	None	Yes	Resistant: Carbapenems, Aminoglycosides
P054	Biopsy	*Klebsiella pneumoniae*	*Bacteroides fragilis*	Yes	Resistant: Beta-lactams, Cephalosporins
P055	Swab	*Enterococcus faecium (VRE)*	None	Yes	Resistant: Vancomycin, Linezolid
P056	Biopsy	*Staphylococcus aureus (MRSA)*	*Peptostreptococcus*	Yes	Resistant: Cephalosporins, Fluoroquinolones
P057	Swab	*Escherichia coli*	None	No	None
P058	Biopsy	*Acinetobacter baumannii*	*Prevotella spp.*	Yes	Resistant: Tigecycline, Colistin
P059	Swab	*Proteus mirabilis*	None	No	None
P060	Biopsy	*Klebsiella pneumoniae (ESBL)*	*Clostridium perfringens*	Yes	Resistant: Carbapenems, Cephalosporins
P061	Swab	*Staphylococcus epidermidis*	None	No	None
P062	Biopsy	*Pseudomonas aeruginosa*	*Bacteroides fragilis*	Yes	Resistant: Carbapenems, Fluoroquinolones
P063	Swab	*Enterobacter cloacae*	None	Yes	Resistant: Cephalosporins, Aminoglycosides
P064	Biopsy	*Staphylococcus aureus (MRSA)*	*Peptostreptococcus*	Yes	Resistant: Fluoroquinolones, Beta-lactams
P065	Swab	*Escherichia coli (ESBL)*	None	Yes	Resistant: Cephalosporins, Fluoroquinolones
P066	Biopsy	*Proteus mirabilis*	*Prevotella spp.*	No	None
P067	Swab	*Pseudomonas aeruginosa*	None	Yes	Resistant: Carbapenems, Aminoglycosides
P068	Biopsy	*Acinetobacter baumannii*	*Clostridium spp*	Yes	Resistant: Colistin, Tigecycline
P069	Swab	*Klebsiella pneumoniae (ESBL)*	None	Yes	Resistant: Cephalosporins, Fluoroquinolones

Patient ID	Sample Collection	Aerobic Culture	Anaerobic Culture	MDR Status	AST (Resistant)
P070	Biopsy	*Enterococcus faecium (VRE)*	*Bacteroides fragilis*	Yes	Resistant: Vancomycin, Linezolid
P071	Swab	*Staphylococcus aureus*	None	No	None
P072	Biopsy	*Pseudomonas aeruginosa*	*Clostridium spp.*	Yes	Resistant: Carbapenems, Beta-lactams
P073	Swab	*Escherichia coli (ESBL)*	None	Yes	Resistant: Cephalosporins, Fluoroquinolones
P074	Biopsy	*Klebsiella pneumoniae*	*Prevotella spp.*	Yes	Resistant: Cephalosporins, Carbapenems
P075	Swab	*Acinetobacter baumannii*	None	Yes	Resistant: Tigecycline, Colistin
P076	Biopsy	*Enterobacter cloacae*	*Clostridium spp.*	Yes	Resistant: Cephalosporins, Aminoglycosides
P077	Swab	*Proteus mirabilis*	None	No	None
P078	Biopsy	*Staphylococcus aureus (MRSA)*	*Bacteroides fragilis*	Yes	Resistant: Cephalosporins, Fluoroquinolones
P079	Swab	*Pseudomonas aeruginosa*	None	Yes	Resistant: Carbapenems, Fluoroquinolones
P080	Biopsy	*Klebsiella pneumoniae (ESBL)*	*Clostridium perfringens*	Yes	Resistant: Cephalosporins, Carbapenems
P081	Swab	*Staphylococcus aureus (MRSA)*	None	Yes	Resistant: Cephalosporins, Fluoroquinolones
P082	Biopsy	*Escherichia coli*	*Bacteroides fragilis*	No	None
P083	Swab	*Pseudomonas aeruginosa*	None	Yes	Resistant: Carbapenems, Aminoglycosides
P084	Biopsy	*Klebsiella pneumoniae (ESBL)*	*Clostridium spp.*	Yes	Resistant: Cephalosporins, Fluoroquinolones
P085	Swab	*Enterococcus faecium (VRE)*	None	Yes	Resistant: Vancomycin, Linezolid
P086	Biopsy	*Proteus mirabilis*	*Prevotella spp.*	No	None
P087	Swab	*Escherichia coli*	None	No	None
P088	Biopsy	*Acinetobacter baumannii*	*Bacteroides fragilis*	Yes	Resistant: Tigecycline, Colistin
P089	Swab	*Staphylococcus epidermidis*	None	No	None
P090	Biopsy	*Pseudomonas aeruginosa*	*Clostridium perfringens*	Yes	Resistant: Carbapenems, Fluoroquinolones
P091	Swab	*Klebsiella pneumoniae*	None	Yes	Resistant: Beta-lactams, Cephalosporins
P092	Biopsy	*Enterobacter cloacae*	*Prevotella spp.*	Yes	Resistant: Carbapenems, Aminoglycosides

Patient ID	Sample Collection	Aerobic Culture	Anaerobic Culture	MDR Status	AST (Resistant)
P093	Swab	*Staphylococcus aureus (MRSA)*	None	Yes	Resistant: Cephalosporins, Fluoroquinolones
P094	Biopsy	*Escherichia coli*	*Peptostreptococcus*	No	None
P095	Swab	*Pseudomonas aeruginosa*	None	Yes	Resistant: Carbapenems, Aminoglycosides
P096	Biopsy	*Klebsiella pneumoniae (ESBL)*	*Clostridium spp.*	Yes	Resistant: Cephalosporins, Beta-lactams
P097	Swab	*Proteus mirabilis*	None	No	None
P098	Biopsy	*Acinetobacter baumannii*	*Prevotella spp.*	Yes	Resistant: Tigecycline, Colistin
P099	Swab	*Enterococcus faecium (VRE)*	None	Yes	Resistant: Vancomycin, Linezolid
P100	Biopsy	*Staphylococcus epidermidis*	*Bacteroides fragilis*	No	None
P101	Swab	*Escherichia coli (ESBL)*	None	Yes	Resistant: Cephalosporins, Fluoroquinolones
P102	Biopsy	*Pseudomonas aeruginosa*	*Clostridium perfringens*	Yes	Resistant: Carbapenems, Aminoglycosides
P103	Swab	*Klebsiella pneumoniae*	None	Yes	Resistant: Cephalosporins, Carbapenems
P104	Biopsy	*Enterobacter cloacae*	*Peptostreptococcus*	Yes	Resistant: Cephalosporins, Aminoglycosides
P105	Swab	*Staphylococcus aureus (MRSA)*	None	Yes	Resistant: Cephalosporins, Fluoroquinolones
P106	Biopsy	*Proteus mirabilis*	*Clostridium spp.*	No	None
P107	Swab	*Acinetobacter baumannii*	None	Yes	Resistant: Colistin, Tigecycline
P108	Biopsy	*Klebsiella pneumoniae (ESBL)*	*Bacteroides fragilis*	Yes	Resistant: Cephalosporins, Fluoroquinolones
P109	Swab	*Escherichia coli*	None	No	None
P110	Biopsy	*Staphylococcus aureus (MRSA)*	*Prevotella spp.*	Yes	Resistant: Cephalosporins, Beta-lactams
P111	Swab	*Escherichia coli (ESBL)*	None	Yes	Resistant: Cephalosporins, Fluoroquinolones
P112	Biopsy	*Staphylococcus aureus*	*Bacteroides fragilis*	No	None
P113	Swab	*Pseudomonas aeruginosa*	None	Yes	Resistant: Carbapenems, Aminoglycosides
P114	Biopsy	*Klebsiella pneumoniae (ESBL)*	*Clostridium spp.*	Yes	Resistant: Cephalosporins, Carbapenems
P115	Swab	*Staphylococcus epidermidis*	None	No	None
P116	Biopsy	*Proteus mirabilis*	*Prevotella spp.*	No	None

Patient ID	Sample Collection	Aerobic Culture	Anaerobic Culture	MDR Status	AST (Resistant)
P117	Swab	*Enterococcus faecium (VRE)*	None	Yes	Resistant: Vancomycin, Linezolid
P118	Biopsy	*Acinetobacter baumannii*	*Peptostreptococcus*	Yes	Resistant: Colistin, Tigecycline
P119	Swab	*Escherichia coli*	None	No	None
P120	Biopsy	*Pseudomonas aeruginosa*	*Clostridium perfringens*	Yes	Resistant: Carbapenems, Cephalosporins
P121	Swab	*Klebsiella pneumoniae*	None	Yes	Resistant: Cephalosporins, Fluoroquinolones
P122	Biopsy	*Enterococcus faecium (VRE)*	*Bacteroides fragilis*	Yes	Resistant: Vancomycin, Linezolid
P123	Swab	*Staphylococcus aureus*	None	No	None
P124	Biopsy	*Escherichia coli*	*Prevotella spp.*	No	None
P125	Swab	*Proteus mirabilis*	None	No	None
P126	Biopsy	*Klebsiella pneumoniae (ESBL)*	*Clostridium spp.*	Yes	Resistant: Cephalosporins, Carbapenems
P127	Swab	*Pseudomonas aeruginosa*	None	Yes	Resistant: Carbapenems, Fluoroquinolones
P128	Biopsy	*Staphylococcus aureus (MRSA)*	*Bacteroides fragilis*	Yes	Resistant: Cephalosporins, Fluoroquinolones
P129	Swab	*Escherichia coli*	None	No	None
P130	Biopsy	*Acinetobacter baumannii*	*Clostridium perfringens*	Yes	Resistant: Tigecycline, Colistin
P131	Swab	*Enterobacter cloacae*	None	Yes	Resistant: Cephalosporins, Aminoglycosides
P132	Biopsy	*Staphylococcus aureus*	*Prevotella spp.*	No	None
P133	Swab	*Pseudomonas aeruginosa*	None	Yes	Resistant: Carbapenems, Aminoglycosides
P134	Biopsy	*Klebsiella pneumoniae (ESBL)*	*Bacteroides fragilis*	Yes	Resistant: Cephalosporins, Carbapenems
P135	Swab	*Staphylococcus epidermidis*	None	No	None
P136	Biopsy	*Acinetobacter baumannii*	*Clostridium spp.*	Yes	Resistant: Tigecycline, Colistin
P137	Swab	*Escherichia coli (ESBL)*	None	Yes	Resistant: Cephalosporins, Fluoroquinolones
P138	Biopsy	*Proteus mirabilis*	*Bacteroides fragilis*	No	None
P139	Swab	*Pseudomonas aeruginosa*	None	Yes	Resistant: Carbapenems, Fluoroquinolones
P140	Biopsy	*Klebsiella pneumoniae (ESBL)*	*Prevotella spp.*	Yes	Resistant: Cephalosporins, Beta-lactams

Patient ID	Sample Collection	Aerobic Culture	Anaerobic Culture	MDR Status	AST (Resistant)
P141	Swab	*Staphylococcus aureus (MRSA)*	None	Yes	Resistant: Cephalosporins, Fluoroquinolones
P142	Biopsy	*Escherichia coli (ESBL)*	*Bacteroides fragilis*	Yes	Resistant: Cephalosporins, Carbapenems
P143	Swab	*Pseudomonas aeruginosa*	None	Yes	Resistant: Carbapenems, Aminoglycosides
P144	Biopsy	*Enterococcus faecium (VRE)*	*Clostridium spp.*	Yes	Resistant: Vancomycin, Linezolid
P145	Swab	*Staphylococcus epidermidis*	None	No	None
P146	Biopsy	*Klebsiella pneumoniae (ESBL)*	*Prevotella spp.*	Yes	Resistant: Cephalosporins, Fluoroquinolones
P147	Swab	*Proteus mirabilis*	None	No	None
P148	Biopsy	*Acinetobacter baumannii*	*Bacteroides fragilis*	Yes	Resistant: Tigecycline, Colistin
P149	Swab	*Escherichia coli*	None	No	None
P150	Biopsy	*Pseudomonas aeruginosa*	*Clostridium perfringens*	Yes	Resistant: Carbapenems, Cephalosporins
P151	Swab	*Klebsiella pneumoniae*	None	Yes	Resistant: Beta-lactams, Carbapenems
P152	Biopsy	*Enterococcus faecium (VRE)*	*Prevotella spp.*	Yes	Resistant: Vancomycin, Linezolid
P153	Swab	*Staphylococcus aureus*	None	No	None
P154	Biopsy	*Escherichia coli*	*Peptostreptococcus*	No	None
P155	Swab	*Pseudomonas aeruginosa*	None	Yes	Resistant: Carbapenems, Fluoroquinolones
P156	Biopsy	*Klebsiella pneumoniae (ESBL)*	*Clostridium spp.*	Yes	Resistant: Cephalosporins, Fluoroquinolones
P157	Swab	*Staphylococcus epidermidis*	None	No	None
P158	Biopsy	*Proteus mirabilis*	*Bacteroides fragilis*	No	None
P159	Swab	*Enterococcus faecium (VRE)*	None	Yes	Resistant: Vancomycin, Linezolid
P160	Biopsy	*Acinetobacter baumannii*	*Prevotella spp.*	Yes	Resistant: Tigecycline, Colistin
P161	Swab	*Staphylococcus aureus (MRSA)*	None	Yes	Resistant: Cephalosporins, Fluoroquinolones
P162	Biopsy	*Pseudomonas aeruginosa*	*Clostridium perfringens*	Yes	Resistant: Carbapenems, Aminoglycosides
P163	Swab	*Escherichia coli (ESBL)*	None	Yes	Resistant: Cephalosporins, Carbapenems

Patient ID	Sample Collection	Aerobic Culture	Anaerobic Culture	MDR Status	AST (Resistant)
P164	Biopsy	*Klebsiella pneumoniae*	*Bacteroides fragilis*	Yes	Resistant: Cephalosporins, Fluoroquinolones
P165	Swab	*Proteus mirabilis*	None	No	None
P166	Biopsy	*Acinetobacter baumannii*	*Peptostreptococcus*	Yes	Resistant: Tigecycline, Colistin
P167	Swab	*Enterobacter cloacae*	None	Yes	Resistant: Cephalosporins, Aminoglycosides
P168	Biopsy	*Staphylococcus aureus*	*Prevotella spp.*	No	None
P169	Swab	*Pseudomonas aeruginosa*	None	Yes	Resistant: Carbapenems, Fluoroquinolones
P170	Biopsy	*Klebsiella pneumoniae (ESBL)*	*Clostridium spp.*	Yes	Resistant: Cephalosporins, Beta-lactams
P171	Swab	*Escherichia coli (ESBL)*	None	Yes	Resistant: Cephalosporins, Fluoroquinolones
P172	Biopsy	*Staphylococcus aureus*	*Bacteroides fragilis*	No	None
P173	Swab	*Pseudomonas aeruginosa*	None	Yes	Resistant: Carbapenems, Aminoglycosides
P174	Biopsy	*Klebsiella pneumoniae (ESBL)*	*Clostridium spp.*	Yes	Resistant: Cephalosporins, Carbapenems
P175	Swab	*Staphylococcus epidermidis*	None	No	None
P176	Biopsy	*Proteus mirabilis*	*Prevotella spp.*	No	None
P177	Swab	*Enterococcus faecium (VRE)*	None	Yes	Resistant: Vancomycin, Linezolid
P178	Biopsy	*Acinetobacter baumannii*	*Bacteroides fragilis*	Yes	Resistant: Tigecycline, Colistin
P179	Swab	*Escherichia coli*	None	No	None
P180	Biopsy	*Pseudomonas aeruginosa*	*Clostridium perfringens*	Yes	Resistant: Carbapenems, Cephalosporins
P181	Swab	*Klebsiella pneumoniae*	None	Yes	Resistant: Beta-lactams, Cephalosporins
P182	Biopsy	*Enterococcus faecium (VRE)*	*Prevotella spp.*	Yes	Resistant: Vancomycin, Linezolid
P183	Swab	*Staphylococcus aureus*	None	No	None
P184	Biopsy	*Escherichia coli*	*Peptostreptococcus*	No	None
P185	Swab	*Pseudomonas aeruginosa*	None	Yes	Resistant: Carbapenems, Fluoroquinolones
P186	Biopsy	*Klebsiella pneumoniae (ESBL)*	*Clostridium spp.*	Yes	Resistant: Cephalosporins, Fluoroquinolones
P187	Swab	*Staphylococcus epidermidis*	None	No	None
P188	Biopsy	*Proteus mirabilis*	*Bacteroides fragilis*	No	None

Patient ID	Sample Collection	Aerobic Culture	Anaerobic Culture	MDR Status	AST (Resistant)
P189	Swab	*Enterococcus faecium (VRE)*	None	Yes	Resistant: Vancomycin, Linezolid
P190	Biopsy	*Acinetobacter baumannii*	*Prevotella spp.*	Yes	Resistant: Tigecycline, Colistin
P191	Swab	*Staphylococcus aureus (MRSA)*	None	Yes	Resistant: Cephalosporins, Fluoroquinolones
P192	Biopsy	*Pseudomonas aeruginosa*	*Clostridium perfringens*	Yes	Resistant: Carbapenems, Aminoglycosides
P193	Swab	*Escherichia coli (ESBL)*	None	Yes	Resistant: Cephalosporins, Carbapenems
P194	Biopsy	*Klebsiella pneumoniae*	*Bacteroides fragilis*	Yes	Resistant: Cephalosporins, Fluoroquinolones
P195	Swab	*Proteus mirabilis*	None	No	None
P196	Biopsy	*Acinetobacter baumannii*	*Peptostreptococcus*	Yes	Resistant: Tigecycline, Colistin
P197	Swab	*Enterobacter cloacae*	None	Yes	Resistant: Cephalosporins, Aminoglycosides
P198	Biopsy	*Staphylococcus aureus (MRSA)*	*Prevotella spp.*	Yes	Resistant: Cephalosporins, Beta-lactams
P199	Swab	*Pseudomonas aeruginosa*	None	Yes	Resistant: Carbapenems, Fluoroquinolones
P200	Biopsy	*Klebsiella pneumoniae (ESBL)*	*Clostridium spp.*	Yes	Resistant: Cephalosporins, Beta-lactams

Supplementary Table 2: Detection of MDR genes in clinical isolates.

S. No.	MDR Isolate	Resistance Gene Detected	Gene Target	PCR Result
1.	*Staphylococcus aureus*	No MDR genes detected	N/A	Negative
2.	*Escherichia coli (ESBL)*	blaCTX-M	*blaCTX-M*	Positive
3.	*Pseudomonas aeruginosa*	blaNDM	*blaNDM*	Positive
4.	*Klebsiella pneumoniae*	blaOXA-48	*blaOXA-48*	Positive
5.	*Streptococcus agalactiae*	No MDR genes detected	N/A	Negative
6.	*Enterococcus faecium (VRE)*	vanA	*vanA*	Positive
7.	*Proteus mirabilis*	No MDR genes detected	N/A	Negative
8.	*Acinetobacter baumannii*	blaNDM	*blaNDM*	Positive
9.	*Enterobacter cloacae*	blaKPC	*blaKPC*	Positive
10.	*Staphylococcus epidermidis*	No MDR genes detected	N/A	Negative
11.	*Staphylococcus aureus (MRSA)*	mecA	*mecA*	Positive
12.	*Escherichia coli*	No MDR genes detected	N/A	Negative
13.	*Proteus mirabilis*	No MDR genes detected	N/A	Negative
14.	*Pseudomonas aeruginosa*	blaOXA-48	*blaOXA-48*	Positive
15.	*Enterococcus faecium (VRE)*	vanB	*vanB*	Positive
16.	*Klebsiella pneumoniae (ESBL)*	blaSHV	*blaSHV*	Positive
17.	*Streptococcus agalactiae*	No MDR genes detected	N/A	Negative
18.	*Acinetobacter baumannii*	blaOXA-48	*blaOXA-48*	Positive
19.	*Enterobacter cloacae*	blaKPC	*blaKPC*	Positive
20.	*Staphylococcus epidermidis*	No MDR genes detected	N/A	Negative
21.	*Escherichia coli (ESBL)*	blaCTX-M	*blaCTX-M*	Positive
22.	*Pseudomonas aeruginosa*	blaNDM	*blaNDM*	Positive
23.	*Proteus mirabilis*	No MDR genes detected	N/A	Negative
24.	*Klebsiella pneumoniae*	blaSHV	*blaSHV*	Positive
25.	*Staphylococcus aureus*	No MDR genes detected	N/A	Negative
26.	*Acinetobacter baumannii*	blaNDM	*blaNDM*	Positive
27.	*Enterococcus faecium (VRE)*	vanA	*vanA*	Positive
28.	*Escherichia coli*	No MDR genes detected	N/A	Negative
29.	*Pseudomonas aeruginosa*	blaOXA-48	*blaOXA-48*	Positive
30.	*Klebsiella pneumoniae (ESBL)*	blaCTX-M	*blaCTX-M*	Positive
31.	*Escherichia coli*	No MDR genes detected	N/A	Negative
32.	*Pseudomonas aeruginosa*	blaKPC	*blaKPC*	Positive
33.	*Staphylococcus aureus (MRSA)*	mecA	*mecA*	Positive
34.	*Enterobacter cloacae*	blaNDM	*blaNDM*	Positive
35.	*Proteus mirabilis*	No MDR genes detected	N/A	Negative
36.	*Klebsiella pneumoniae (ESBL)*	blaSHV	*blaSHV*	Positive
37.	*Streptococcus agalactiae*	No MDR genes detected	N/A	Negative
38.	*Acinetobacter baumannii*	blaOXA-48	*blaOXA-48*	Positive
39.	*Enterococcus faecium (VRE)*	vanA	*vanA*	Positive
40.	*Escherichia coli (ESBL)*	blaCTX-M	*blaCTX-M*	Positive
41.	*Pseudomonas aeruginosa*	blaNDM	*blaNDM*	Positive
42.	*Staphylococcus epidermidis*	No MDR genes detected	N/A	Negative
43.	*Klebsiella pneumoniae*	blaSHV	*blaSHV*	Positive
44.	*Acinetobacter baumannii*	blaNDM	*blaNDM*	Positive
45.	*Proteus mirabilis*	No MDR genes detected	N/A	Negative
46.	*Enterococcus faecium (VRE)*	vanB	*vanB*	Positive
47.	*Escherichia coli*	No MDR genes detected	N/A	Negative
48.	*Staphylococcus aureus (MRSA)*	mecA	*mecA*	Positive
49.	*Pseudomonas aeruginosa*	blaOXA-48	*blaOXA-48*	Positive
50.	*Klebsiella pneumoniae (ESBL)*	blaSHV	*blaSHV*	Positive
51.	*Staphylococcus aureus*	No MDR genes detected	N/A	Negative
52.	*Escherichia coli (ESBL)*	blaTEM	*blaTEM*	Positive
53.	*Pseudomonas aeruginosa*	blaNDM	*blaNDM*	Positive
54.	*Klebsiella pneumoniae*	blaOXA-48	*blaOXA-48*	Positive

S. No.	MDR Isolate	Resistance Gene Detected	Gene Target	PCR Result
55.	*Enterococcus faecium (VRE)*	vanA	*vanA*	Positive
56.	*Staphylococcus aureus (MRSA)*	mecA	*mecA*	Positive
57.	*Escherichia coli*	No MDR genes detected	N/A	Negative
58.	*Acinetobacter baumannii*	blaOXA-48	*blaOXA-48*	Positive
59.	*Proteus mirabilis*	No MDR genes detected	N/A	Negative
60.	*Klebsiella pneumoniae (ESBL)*	blaSHV	*blaSHV*	Positive
61.	*Staphylococcus epidermidis*	No MDR genes detected	N/A	Negative
62.	*Pseudomonas aeruginosa*	blaNDM	*blaNDM*	Positive
63.	*Enterobacter cloacae*	blaKPC	*blaKPC*	Positive
64.	*Staphylococcus aureus (MRSA)*	mecA	*mecA*	Positive
65.	*Escherichia coli (ESBL)*	blaCTX-M	*blaCTX-M*	Positive
66.	*Proteus mirabilis*	No MDR genes detected	N/A	Negative
67.	*Pseudomonas aeruginosa*	blaOXA-48	*blaOXA-48*	Positive
68.	*Acinetobacter baumannii*	blaNDM	*blaNDM*	Positive
69.	*Klebsiella pneumoniae (ESBL)*	blaSHV	*blaSHV*	Positive
70.	*Enterococcus faecium (VRE)*	vanA	*vanA*	Positive
71.	*Staphylococcus aureus*	No MDR genes detected	N/A	Negative
72.	*Pseudomonas aeruginosa*	blaOXA-48	*blaOXA-48*	Positive
73.	*Escherichia coli (ESBL)*	blaSHV	*blaSHV*	Positive
74.	*Klebsiella pneumoniae*	blaOXA-48	*blaOXA-48*	Positive
75.	*Acinetobacter baumannii*	blaNDM	*blaNDM*	Positive
76.	*Enterobacter cloacae*	blaKPC	*blaKPC*	Positive
77.	*Proteus mirabilis*	No MDR genes detected	N/A	Negative
78.	*Staphylococcus aureus (MRSA)*	mecA	*mecA*	Positive
79.	*Pseudomonas aeruginosa*	blaNDM	*blaNDM*	Positive
80.	*Klebsiella pneumoniae (ESBL)*	blaCTX-M	*blaCTX-M*	Positive
81.	*Staphylococcus aureus (MRSA)*	mecA	*mecA*	Positive
82.	*Escherichia coli*	No MDR genes detected	N/A	Negative
83.	*Pseudomonas aeruginosa*	blaNDM	*blaNDM*	Positive
84.	*Klebsiella pneumoniae (ESBL)*	blaSHV	*blaSHV*	Positive
85.	*Enterococcus faecium (VRE)*	vanB	*vanB*	Positive
86.	*Proteus mirabilis*	No MDR genes detected	N/A	Negative
87.	*Escherichia coli*	No MDR genes detected	N/A	Negative
88.	*Acinetobacter baumannii*	blaOXA-48	*blaOXA-48*	Positive
89.	*Staphylococcus epidermidis*	No MDR genes detected	N/A	Negative
90.	*Pseudomonas aeruginosa*	blaOXA-48	*blaOXA-48*	Positive
91.	*Klebsiella pneumoniae*	blaSHV	*blaSHV*	Positive
92.	*Enterobacter cloacae*	blaKPC	*blaKPC*	Positive
93.	*Staphylococcus aureus (MRSA)*	mecA	*mecA*	Positive
94.	*Escherichia coli*	No MDR genes detected	N/A	Negative
95.	*Pseudomonas aeruginosa*	blaOXA-48	*blaOXA-48*	Positive
96.	*Klebsiella pneumoniae (ESBL)*	blaSHV	*blaSHV*	Positive
97.	*Proteus mirabilis*	No MDR genes detected	N/A	Negative
98.	*Acinetobacter baumannii*	blaNDM	*blaNDM*	Positive
99.	*Enterococcus faecium (VRE)*	vanA	*vanA*	Positive
100.	*Staphylococcus epidermidis*	No MDR genes detected	N/A	Negative
101.	*Escherichia coli (ESBL)*	blaCTX-M	*blaCTX-M*	Positive
102.	*Pseudomonas aeruginosa*	blaNDM	*blaNDM*	Positive
103.	*Klebsiella pneumoniae*	blaSHV	*blaSHV*	Positive
104.	*Enterobacter cloacae*	blaKPC	*blaKPC*	Positive
105.	*Staphylococcus aureus (MRSA)*	mecA	*mecA*	Positive
106.	*Proteus mirabilis*	No MDR genes detected	N/A	Negative
107.	*Acinetobacter baumannii*	blaOXA-48	*blaOXA-48*	Positive
108.	*Klebsiella pneumoniae (ESBL)*	blaSHV	*blaSHV*	Positive
109.	*Escherichia coli*	No MDR genes detected	N/A	Negative
110.	*Staphylococcus aureus (MRSA)*	mecA	*mecA*	Positive
111.	*Escherichia coli (ESBL)*	blaCTX-M	*blaCTX-M*	Positive

S. No.	MDR Isolate	Resistance Gene Detected	Gene Target	PCR Result
112.	*Staphylococcus aureus*	No MDR genes detected	N/A	Negative
113.	*Pseudomonas aeruginosa*	blaNDM	*blaNDM*	Positive
114.	*Klebsiella pneumoniae (ESBL)*	blaSHV	*blaSHV*	Positive
115.	*Staphylococcus epidermidis*	No MDR genes detected	N/A	Negative
116.	*Proteus mirabilis*	No MDR genes detected	N/A	Negative
117.	*Enterococcus faecium (VRE)*	vanB	*vanB*	Positive
118.	*Acinetobacter baumannii*	blaNDM	*blaNDM*	Positive
119.	*Escherichia coli*	No MDR genes detected	N/A	Negative
120.	*Pseudomonas aeruginosa*	blaOXA-48	*blaOXA-48*	Positive
121.	*Klebsiella pneumoniae*	blaSHV	*blaSHV*	Positive
122.	*Enterococcus faecium (VRE)*	vanA	*vanA*	Positive
123.	*Staphylococcus aureus*	No MDR genes detected	N/A	Negative
124.	*Escherichia coli*	No MDR genes detected	N/A	Negative
125.	*Proteus mirabilis*	No MDR genes detected	N/A	Negative
126.	*Klebsiella pneumoniae (ESBL)*	blaCTX-M	*blaCTX-M*	Positive
127.	*Pseudomonas aeruginosa*	blaNDM	*blaNDM*	Positive
128.	*Staphylococcus aureus (MRSA)*	mecA	*mecA*	Positive
129.	*Escherichia coli*	No MDR genes detected	N/A	Negative
130.	*Acinetobacter baumannii*	blaOXA-48	*blaOXA-48*	Positive
131.	*Enterobacter cloacae*	blaKPC	*blaKPC*	Positive
132.	*Staphylococcus aureus*	No MDR genes detected	N/A	Negative
133.	*Pseudomonas aeruginosa*	blaNDM	*blaNDM*	Positive
134.	*Klebsiella pneumoniae (ESBL)*	blaSHV	*blaSHV*	Positive
135.	*Staphylococcus epidermidis*	No MDR genes detected	N/A	Negative
136.	*Acinetobacter baumannii*	blaOXA-48	*blaOXA-48*	Positive
137.	*Escherichia coli (ESBL)*	blaCTX-M	*blaCTX-M*	Positive
138.	*Proteus mirabilis*	No MDR genes detected	N/A	Negative
139.	*Pseudomonas aeruginosa*	blaNDM	*blaNDM*	Positive
140.	*Klebsiella pneumoniae (ESBL)*	blaCTX-M	*blaCTX-M*	Positive
141.	*Staphylococcus aureus (MRSA)*	mecA	*mecA*	Positive
142.	*Escherichia coli (ESBL)*	blaSHV	*blaSHV*	Positive
143.	*Pseudomonas aeruginosa*	blaOXA-48	*blaOXA-48*	Positive
144.	*Enterococcus faecium (VRE)*	vanA	*vanA*	Positive
145.	*Staphylococcus epidermidis*	No MDR genes detected	N/A	Negative
146.	*Klebsiella pneumoniae (ESBL)*	blaCTX-M	*blaCTX-M*	Positive
147.	*Proteus mirabilis*	No MDR genes detected	N/A	Negative
148.	*Acinetobacter baumannii*	blaOXA-48	*blaOXA-48*	Positive
149.	*Escherichia coli*	No MDR genes detected	N/A	Negative
150.	*Pseudomonas aeruginosa*	blaNDM	*blaNDM*	Positive
151.	*Klebsiella pneumoniae*	blaSHV	*blaSHV*	Positive
152.	*Enterococcus faecium (VRE)*	vanB	*vanB*	Positive
153.	*Staphylococcus aureus*	No MDR genes detected	N/A	Negative
154.	*Escherichia coli*	No MDR genes detected	N/A	Negative
155.	*Pseudomonas aeruginosa*	blaNDM	*blaNDM*	Positive
156.	*Klebsiella pneumoniae (ESBL)*	blaCTX-M	*blaCTX-M*	Positive
157.	*Staphylococcus epidermidis*	No MDR genes detected	N/A	Negative
158.	*Proteus mirabilis*	No MDR genes detected	N/A	Negative
159.	*Enterococcus faecium (VRE)*	vanA	*vanA*	Positive
160.	*Acinetobacter baumannii*	blaOXA-48	*blaOXA-48*	Positive
161.	*Staphylococcus aureus (MRSA)*	mecA	*mecA*	Positive
162.	*Pseudomonas aeruginosa*	blaOXA-48	*blaOXA-48*	Positive
163.	*Escherichia coli (ESBL)*	blaSHV	*blaSHV*	Positive
164.	*Klebsiella pneumoniae (ESBL)*	blaCTX-M	*blaCTX-M*	Positive
165.	*Proteus mirabilis*	No MDR genes detected	N/A	Negative
166.	*Acinetobacter baumannii*	blaNDM	*blaNDM*	Positive
167.	*Enterobacter cloacae*	blaKPC	*blaKPC*	Positive
168.	*Staphylococcus aureus*	No MDR genes detected	N/A	Negative

S. No.	MDR Isolate	Resistance Gene Detected	Gene Target	PCR Result
169.	*Pseudomonas aeruginosa*	blaNDM	*blaNDM*	Positive
170.	*Klebsiella pneumoniae (ESBL)*	blaSHV	*blaSHV*	Positive
171.	*Escherichia coli (ESBL)*	blaCTX-M	*blaCTX-M*	Positive
172.	*Staphylococcus aureus*	No MDR genes detected	N/A	Negative
173.	*Pseudomonas aeruginosa*	blaNDM	*blaNDM*	Positive
174.	*Klebsiella pneumoniae (ESBL)*	blaSHV	*blaSHV*	Positive
175.	*Staphylococcus epidermidis*	No MDR genes detected	N/A	Negative
176.	*Proteus mirabilis*	No MDR genes detected	N/A	Negative
177.	*Enterococcus faecium (VRE)*	vanA	*vanA*	Positive
178.	*Acinetobacter baumannii*	blaOXA-48	*blaOXA-48*	Positive
179.	*Escherichia coli*	No MDR genes detected	N/A	Negative
180.	*Pseudomonas aeruginosa*	blaOXA-48	*blaOXA-48*	Positive
181.	*Klebsiella pneumoniae*	blaSHV	*blaSHV*	Positive
182.	*Enterococcus faecium (VRE)*	vanA	*vanA*	Positive
183.	*Staphylococcus aureus*	No MDR genes detected	N/A	Negative
184.	*Escherichia coli*	No MDR genes detected	N/A	Negative
185.	*Pseudomonas aeruginosa*	blaNDM	*blaNDM*	Positive
186.	*Klebsiella pneumoniae (ESBL)*	blaCTX-M	*blaCTX-M*	Positive
187.	*Staphylococcus epidermidis*	No MDR genes detected	N/A	Negative
188.	*Proteus mirabilis*	No MDR genes detected	N/A	Negative
189.	*Enterococcus faecium (VRE)*	vanB	*vanB*	Positive
190.	*Acinetobacter baumannii*	blaOXA-48	*blaOXA-48*	Positive
191.	*Staphylococcus aureus (MRSA)*	mecA	*mecA*	Positive
192.	*Pseudomonas aeruginosa*	blaOXA-48	*blaOXA-48*	Positive
193.	*Escherichia coli (ESBL)*	blaCTX-M	*blaCTX-M*	Positive
194.	*Klebsiella pneumoniae*	blaSHV	*blaSHV*	Positive
195.	*Proteus mirabilis*	No MDR genes detected	N/A	Negative
196.	*Acinetobacter baumannii*	blaNDM	*blaNDM*	Positive
197.	*Enterobacter cloacae*	blaKPC	*blaKPC*	Positive
198.	*Staphylococcus aureus (MRSA)*	mecA	*mecA*	Positive
199.	*Pseudomonas aeruginosa*	blaNDM	*blaNDM*	Positive
200.	*Klebsiella pneumoniae (ESBL)*	blaCTX-M	*blaCTX-M*	Positive
